# Intratumoral aluminum hydroxide–anchored IL-12 drives potent antitumor activity by remodeling the tumor microenvironment

**DOI:** 10.1172/jci.insight.168224

**Published:** 2023-12-08

**Authors:** Sailaja Battula, Gregory Papastoitsis, Howard L. Kaufman, K. Dane Wittrup, Michael M. Schmidt

**Affiliations:** 1 Ankyra Therapeutics, Boston, Massachusetts, USA.; 2 Massachusetts Institute of Technology, Cambridge, Massachusetts, USA.

**Keywords:** Immunology, Oncology, Cancer immunotherapy, Cytokines

## Abstract

IL-12 is a potent cytokine that can promote innate and adaptive anticancer immunity, but its clinical development has been limited by toxicity when delivered systemically. Intratumoral (i.t.) administration can expand the therapeutic window of IL-12 and other cytokines but is in turn limited by rapid drug clearance from the tumor, which reduces efficacy, necessitates frequent administration, and increases systemic accumulation. To address these limitations, we developed an anchored IL-12 designated ANK-101, composed of an engineered IL-12 variant that forms a stable complex with the FDA-approved vaccine adjuvant aluminum hydroxide (Alhydrogel). Following i.t. administration of murine ANK-101 (mANK-101) in early intervention syngeneic mouse tumors, the complex formed a depot that was locally retained for weeks as measured by IVIS or SPECT/CT imaging, while unanchored protein injected i.t. was cleared within hours. One or 2 i.t. injections of mANK-101 induced single-agent antitumor activity across a diverse range of syngeneic tumors, including models resistant to checkpoint blockade at doses where unanchored IL-12 had no efficacy. Local treatment with mANK-101 further induced regressions of noninjected lesions, especially when combined with systemic checkpoint blockade. Antitumor activity was associated with remodeling of the tumor microenvironment, including prolonged IFN-γ and chemokine expression, recruitment and activation of T and NK cells, M1 myeloid cell skewing, and increased antigen processing and presentation. Subcutaneous administration of ANK-101 in cynomolgus macaques was well tolerated. Together, these data demonstrate that ANK-101 has an enhanced efficacy and safety profile and warrants future clinical development.

## Graphical abstract

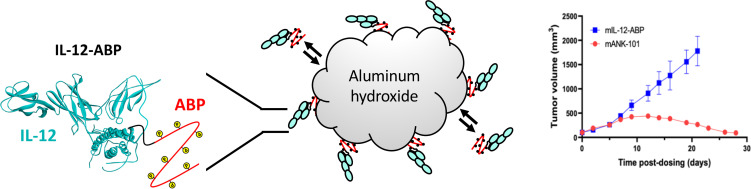

## Introduction

While immune checkpoint inhibitors targeting PD-1/PD-L1 have been transformative therapies for many cancers, innate and acquired resistance remains a challenge (1). Recombinant cytokines have potential to improve responses in these immunologically resistant tumors by directly promoting recruitment and activation of effector immune cell populations (2, 3). IL-12 is a heterodimeric cytokine composed of p35 and p40 subunits that signals through the IL-12RB1/IL-12RB2 receptor complex on T and NK cells to induce IFN-γ expression and Th1 polarization (4). Through a combination of direct signaling and indirect effects of IFN-γ upregulation, IL-12 can induce immune cell recruitment, costimulation, myeloid cell repolarization, and antigen presentation (5).

Treatment with recombinant IL-12 either as a single agent or in combination with other immunotherapies has demonstrated profound efficacy in a variety of preclinical models, including tumors resistant to checkpoint blockade (6, 7). However, clinical translation of IL-12 has been hindered by severe dose-limiting toxicities when delivered intravenously (i.v.) or subcutaneously (s.c.), including lymphopenia, hepatotoxicity, and gastrointestinal inflammation leading to deaths of 2 patients in early clinical trials (8–10). Due to these systemic toxicities, only very low doses of IL-12 in the range of 0.3–1 μg/kg could be safely administered in clinical studies, limiting tumor accumulation and efficacy (11, 12). There is, therefore, a need to expand the therapeutic window of IL-12 and other cytokines by increasing their relative tumor concentration while reducing systemic exposure and associated toxicity.

Intratumoral (i.t.) administration can improve the therapeutic window of drugs by delivering them directly into the tumor (13–16). While i.t. administration has historically been limited to surface-accessible lesions, advances in interventional radiology and surgical techniques have made nearly all tumors accessible for injection (17). Recent clinical studies have demonstrated that i.t. administration of immune-stimulating agents can induce regressions of both injected and noninjected lesions through priming of a systemic immune response, especially when combined with checkpoint blockade (18–21). However, the potential benefits of i.t. administration remain limited by rapid clearance of injected drug from the tumor through vascular intravasation and lymphatic drainage (22, 23). Such clearance limits local drug exposure, thereby reducing efficacy or necessitating frequent administrations, as well as increases systemic exposure and potential toxicity. Previous clinical attempts to deliver unmodified IL-12 intratumorally had limited efficacy and significant toxicity, likely due to rapid drug clearance from the tumor (24).

Multiple approaches have been described recently to enhance the tumor residence of IL-12 and other cytokines, including fusion to tumor-targeting antibodies (25, 26), binding to extracellular matrix (ECM) components (22, 27), increased size (28), coformulation with biodegradable matrices (29), or local expression from DNA, mRNA, or oncolytic viruses (30–32). Such approaches have demonstrated enhanced tolerability and activity compared with unmodified IL-12 in preclinical and in some cases clinical studies. Despite these advances, many of these technologies remain suboptimal, with only modest increases in tumor retention, unpredictable dosing due to heterogeneous IL-12 expression, or variable retention due to differences in tumor antigen and ECM target levels across individual patients and tumors.

An approach to enhance tumor retention of therapeutic proteins called “anchored immunotherapy” was recently described in which cytokines are stably complexed with the US Food and Drug Administration–approved (FDA-approved) vaccine adjuvant aluminum hydroxide (Alhydrogel) (33, 34). Aluminum hydroxide is composed of highly charged, micron-sized aggregates that form a physical depot at the injection site that can be locally retained for several weeks (35, 36). Cytokines or therapeutic proteins can be fused to an alum-binding peptide (ABP) that becomes phosphorylated on multiple serines through coexpression with the kinase Fam20C (37). When mixed with aluminum hydroxide, the phosphorylated peptide undergoes a ligand-exchange reaction with surface hydroxyl groups on the particles, leading to stable tethering of the cytokine payload (38, 39). These complexes are delivered intratumorally where they form a depot, leading to prolonged and enhanced immune activation at the injected site. Here, we characterize an optimized IL-12-ABP/Alhydrogel complex referred to as ANK-101, including quantification of retention and biodistribution following i.t. administration, comprehensive profiling of changes to the tumor microenvironment (TME), optimization of antitumor activity against a range of preclinical tumor models, and demonstration of safety in nonhuman primates.

## Results

### IL-12-ABP protein forms a stable complex with Alhydrogel and maintains activity.

**Figure 1:**
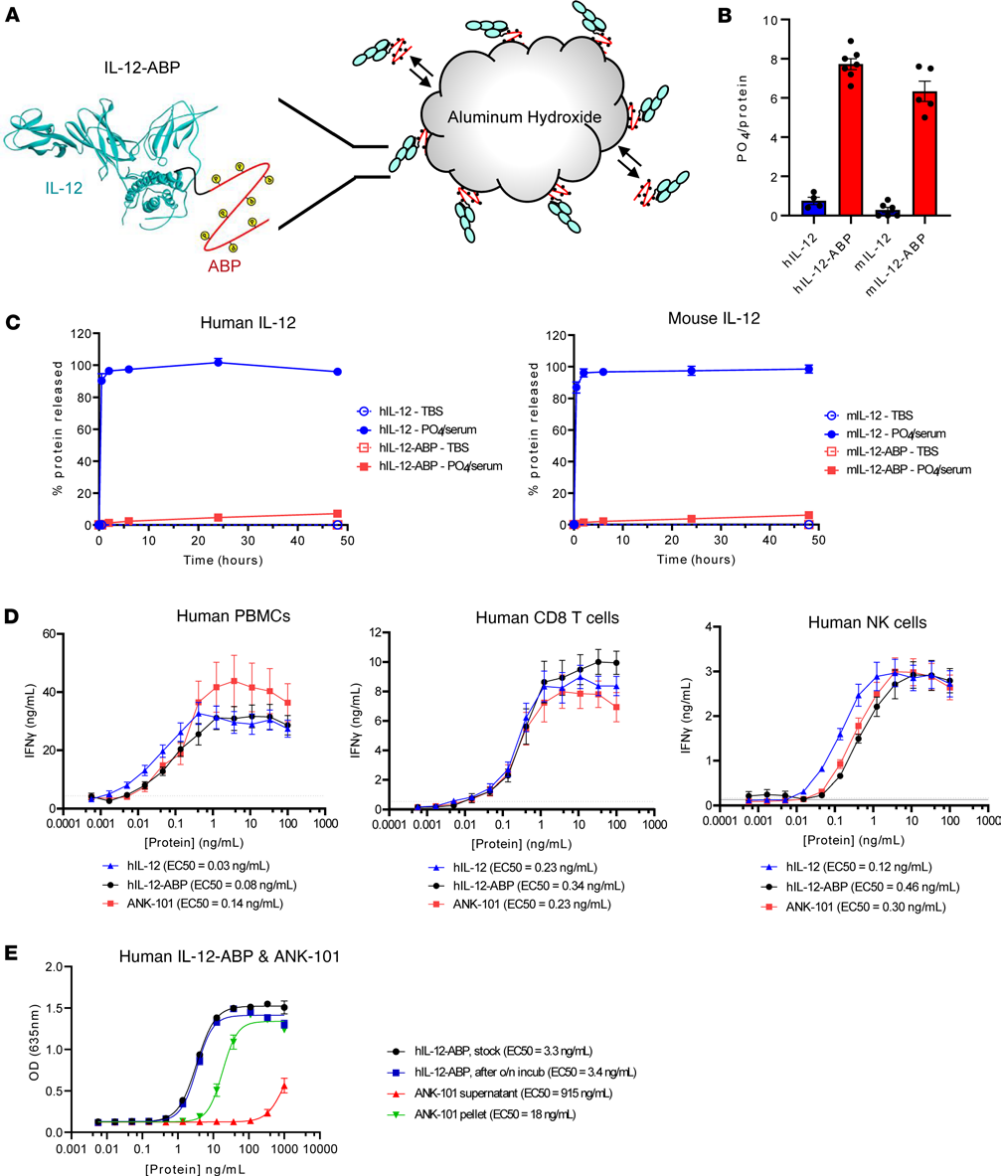
Phosphorylated IL-12-ABP proteins form stable complex with aluminum hydroxide and retain functional activity. (**A**) Schematic of IL-12-ABP fusion protein forming complex with aluminum hydroxide particles through phosphorylated alum-binding peptide (ABP). (**B**) Average number of phosphate molecules per human or mouse IL-12-ABP fusion protein as measured by malachite green assay compared to wild-type IL-12 controls (mean of at least 4 independent experiments ± SD). (**C**) Percentage of Alhydrogel-complexed IL-12-ABP or wild-type IL-12 protein released into supernatant during incubation in Tris-buffered saline (TBS) or 40% serum and 1 mM phosphate, as measured by ELISA (*n* = 3, mean ± SD). Shown are human (left) and mouse (right) assays. (**D**) IFN-γ production measured by TR-FRET from activated human PBMCs (left), purified CD8^+^ T cells (center), or NK cells (right) following 3-day incubation with a titration of wild-type IL-12, free IL-12-ABP protein, or ANK-101 complex (mean of 5 donors ± SEM). (**E**) IL-12 signaling activity measured by HEK-Blue IL-12 assay of resuspended pellets of ANK-101 complex compared to supernatant following complex incubation in 1 mM phosphate and 20% serum for 24 hours (mean of 3 independent experiments ± SD).

ANK-101 is composed of a single-chain human IL-12 protein fused to a C-terminal, phosphorylated ABP (IL-12-ABP) complexed with Alhydrogel (Figure 1A). Gene constructs were generated encoding human IL-12β (p40) and IL-12α (p35) connected through a (G_4_S)_3_ linker either with or without C-terminal fusion to an ABP containing 8 repeats of the sequence motif (SEEGGGG). This ABP was selected, as it had greater alum retention and improved antitumor activity compared with the previously reported ABP construct with 10 repeats (data not shown) (33). Since human IL-12 is not cross-reactive in mice, the surrogate mouse construct mIL-12-ABP containing the murine p40 and p35 sequences was generated for preclinical development. Constructs were coexpressed with Fam20C kinase, which phosphorylates the amino acid motif SEE at multiple sites in the ABP. IL-12-ABP proteins were reproducibly phosphorylated with a mean of 8 (human) or 6 (mouse) phosphates, as measured by the malachite green assay (Figure 1B and Supplemental Figure 1A; supplemental material available online with this article; https://doi.org/10.1172/jci.insight.168224DS1).

When mixed with a 10-fold mass excess of Alhydrogel in Tris-buffered saline (TBS), both wild-type IL-12 and IL-12-ABP proteins bound to the particles through electrostatic interactions between the negatively charged IL-12-ABP and positively charged aluminum hydroxide. Following exposure to phosphate and serum to model physiological conditions, the wild-type IL-12 proteins were rapidly eluted, with greater than 95% in the supernatant by 30 minutes, while the IL-12-ABP proteins remained tightly bound with greater than 90% still complexed at 48 hours (Figure 1C). This enhanced retention depends on both the ABP fusion and coexpression with Fam20C kinase (Supplemental Figure 1A). This dual dependence confirms that increased complex stability is mediated by phosphoserines in the ABP undergoing ligand-exchange reactions with the aluminum hydroxide.

To determine whether the ABP fusion or complexation with aluminum hydroxide impacts biological activity, wild-type human IL-12, unanchored IL-12-ABP, or ANK-101 complex was titrated on activated, primary human PBMCs, CD8^+^ T cells, or NK cells. All 3 molecules induced potent IFN-γ expression from each cell type, with a small increase in EC_50_ for hIL-12-ABP and hANK-101 compared with wild-type hIL-12 on PBMCs and NK cells only, potentially due to steric hindrance or electrostatic repulsion from the ABP fusion (Figure 1D). Importantly, there was no difference in potency between the free and Alhydrogel-complexed forms of IL-12-ABP, indicating that the cytokine remains accessible and active after immobilization (Figure 1E). Similar EC_50_ values were measured for unanchored murine IL-12-ABP and mANK-101 on activated, murine splenocytes, supporting their use for preclinical studies (Supplemental Figure 1B). Both ANK-101 and mANK-101 complexes retained biological activity after extended incubation in phosphate/serum and centrifugation to remove unbound protein, thus highlighting the stability of the complex (Figure 1E).

### mANK-101 complexes are retained in the tumor after i.t. administration.

**Figure 2:**
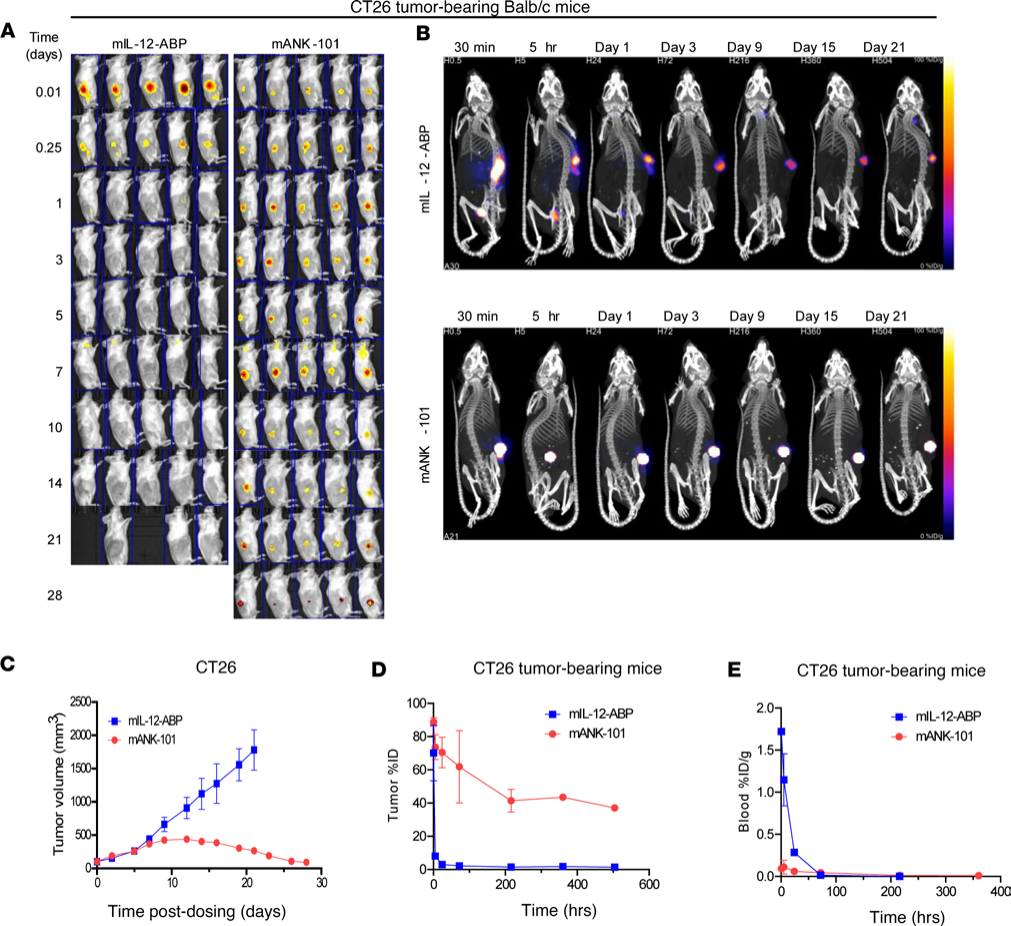
mANK-101 is retained in the tumor after i.t. administration, with reduced systemic exposure. (**A**) IVIS images and (**B**) tumor volumes from BALB/c mice bearing CT26 tumors (mean TV = 101 mm^3^) following a single i.t. administration of Alexa Fluor 647–conjugated mIL-12-ABP as free protein or mANK-101 complex (*n* = 5, mean ± SEM). (**C**) Representative SPECT/CT images of BALB/c mice bearing CT26 tumors after a single i.t. administration of ^125^I-labeled mIL-12-ABP as free protein or mANK-101 complex. (**D**) Percentage injected dose (%ID) in tumor or (**E**) percentage injected dose per gram (%ID/g) in blood of ^125^I-labeled mIL-12-ABP or mANK-101, as measured from SPECT/CT image analysis or ex vivo gamma counts from whole blood, respectively (mean ± SEM, *n* = 3 through the 216-hour time point).

Tumor retention of Alexa Fluor 647–conjugated mIL-12-ABP protein either alone or after Alhydrogel complexation to form mANK-101 was assessed by in vivo IVIS imaging. Following a single i.t. injection in BALB/c mice bearing CT26 tumors, unanchored mIL-12-ABP protein rapidly escaped from the tumor, with minimal signal detected by 24 hours, while mANK-101 was highly retained and still detectable at study completion on day 28 (Figure 2A). Notably, the extended i.t. retention of the mANK-101 complex significantly enhanced antitumor efficacy, as all 5 mice treated with mANK-101 had tumor regressions on day 28, while tumors in free mIL-12-ABP–treated animals exceeded the 2000 mm^3^ humane endpoint by day 23 (Figure 2B).

To measure mANK-101 biodistribution more quantitatively, a SPECT/CT imaging study was performed in which mIL-12-ABP protein was labeled with ^125^I radioisotope (40). ^125^I is a nonresidualizing radioisotope such that detected signal should represent intact protein rather than intracellularly retained radiolabel (41). Following i.t. injection in CT26 tumors, most mANK-101 was retained at the injection site for at least 21 days, whereas most of the free IL-12-ABP was not retained beyond day 1 (Figure 2C and Supplemental Figure 2A). Quantifying this difference by SPECT/CT and ex vivo gamma counts, a small residual signal for free IL-12-ABP remained on day 21, representing approximately 1% or 0.1% of the total percentage injected dose (%ID) (Supplemental Figure 2, A and B). In contrast, the mANK-101 complex was highly retained in the tumor on day 21, with 37% or 31% of the total %ID remaining on day 21, as measured by SPECT/CT or ex vivo gamma count, respectively. Free ^125^I-mIL-12-ABP protein was rapidly cleared from the tumor, with greater than 90% of the injected dose (%ID) eliminated by 5 hours (Figure 2D). The ^125^I signal in serial whole blood samples from animals treated with free mIL-12-ABP increased rapidly after i.t. administration, with a *t*_max_ of 30 minutes and *C*_max_ of 1.7%ID/g (Figure 2E). In contrast, peak ^125^I levels in the blood from mANK-101–treated animals were approximately 16-fold lower, consistent with reduced systemic accumulation due to greater local retention of the complex. SPECT/CT imaging of peripheral tissues detected ^125^I signal in kidney, liver, bladder, and thyroid for free mIL-12-ABP but no significant signal outside of the tumor for mANK-101, except for low bladder levels at 5 and 24 hours (Supplemental Figure 2C).

### mANK-101 induces potent antitumor activity against injected and noninjected lesions.

**Figure 3:**
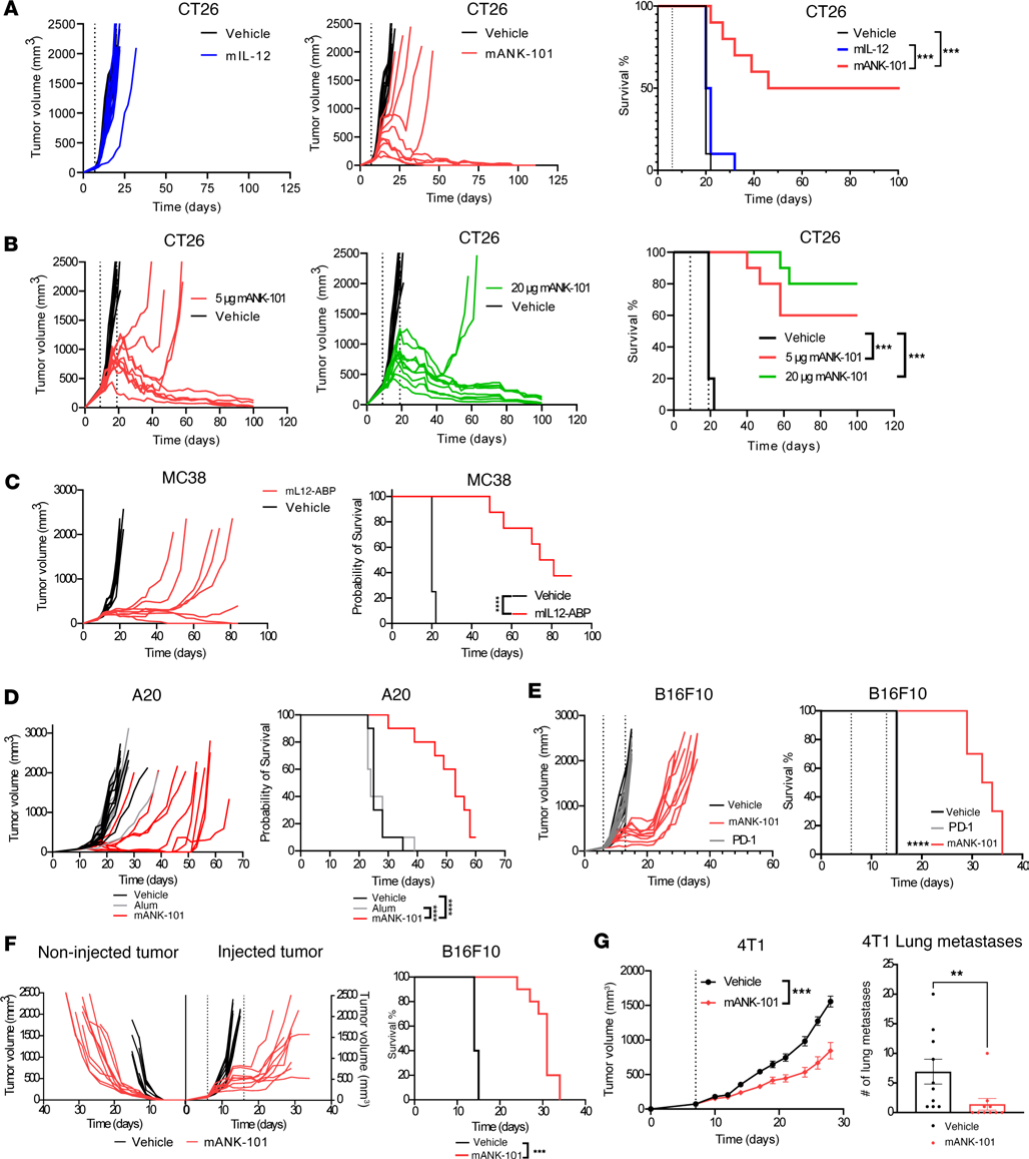
Antitumor activity of mANK-101 in vivo. (**A**) Tumor growth from mice (*n* = 10) bearing CT26 tumors (mean TV = 79 mm^3^) treated with a single i.t. injection of vehicle, 5 μg mIL-12 (left), or 5 μg mANK-101 (center) and survival (right). The dashed vertical line denotes the treatment day. (**B**) Tumor growth from mice (*n* = 10) bearing CT26 tumors (mean TV = 285 mm^3^) treated with 2 i.t. injections of vehicle, 5 (left), or 20 μg mANK-101 (center) 10 days apart, and survival (right). (**C**) Tumor growth from mice (*n* = 8) bearing MC38 tumors treated with a single i.t. injection of vehicle, 5 μg mANK-101 (left), and survival (right). (**D**) Tumor growth from mice (*n* = 10) bearing A20 tumors treated with 2 i.t. injections of 5 μg mANK-101, vehicle, or 50 μg Alhydrogel spaced 7 days apart (left) and survival (right). (**E**) Tumor growth from mice (*n* = 10) bearing B16F10 tumors treated with 2 i.t. injections of 7.5 μg mANK-101 on days 6 and 13 or anti–PD-1 antibody on days 6, 9, 12, and 15 or vehicle (left) and survival (right). (**F**) Tumor growth (left) and survival (right) from mice (*n* = 10) bearing dual-flank B16F10 tumors treated with 2 i.t. injections of vehicle or mANK-101 10 days apart in the right flank tumor only. (**G**) Tumor growth (left) and lung metastases count (right) from mice (*n* = 10) bearing orthotopic 4T-1 tumors (mean TV = 72 mm^3^) treated with 1 i.t. injection of vehicle or 5 μg mANK-101. Animals were sacrificed and lung metastases counted on day 28. Survival in all the efficacy studies utilized the Kaplan-Meier method and was compared by log-rank Mantel-Cox test. 4T-1 mean tumor volume and lung metastases count on day 28 were compared by 1-way ANOVA with Dunnett’s post hoc test. **P < 0.005; ***P < 0.0005; ****P < 0.0001.

The antitumor activity of i.t. mANK-101 was then assessed in vivo using an early intervention model in murine CT26 colorectal cancer. We first tested i.t. mANK-101 in BALB/c mice bearing approximately 80-mm^3^ CT26 tumors. A single i.t. injection of 5 μg mANK-101 resulted in a tumor-free survival of 50%, while an equivalent i.t. dose of wild-type mIL-12 or vehicle had no antitumor efficacy (Figure 3A). A dose response was observed in this model with partial, but reduced, efficacy with a single 2 μg dose of mANK-101 relative to a 5 μg dose, and no further therapeutic benefit from escalating the dose from 5 μg to 10 μg (Supplemental Figure 3A). Two i.t. injections of 5 μg mANK-101 one week apart increased tumor-free survival to 80%; however, no further survival benefit was provided by additional injections every 7, 10, or 14 days (Supplemental Figure 3, B and C). Notably, mANK-101 injected s.c. on the opposite flank had no therapeutic benefit, highlighting the importance of i.t. administration (Supplemental Figure 3B). mANK-101 was also efficacious against large CT26 tumors with a mean tumor volume of 285 mm^3^, with 2 i.t. injections of 5 or 20 μg mANK-101 leading to tumor-free survival rates more than 100 days of 60% or 80%, respectively (Figure 3B). Based on these data, the 5 μg dose was selected as the optimal dose for further testing. To demonstrate that the mANK-101 immune activation was localized to the TME, we collected tumors, tumor-draining lymph nodes, splenocytes, and peripheral blood samples from treated mice on day 7 and performed flow cytometry. We found an increased CD8^+^ cell/Treg ratio only in the TME (Supplemental Figure 3D).

We next evaluated mANK-101 in other syngeneic tumor models with variable immunogenicity, including MC38, A20, B16F10, and 4T1. We tested mANK-101 activity in an MC38 colon cancer model in C57BL/6 mice and saw responses similar to those in the CT26 model (Figure 3C). Next, we tested mANK-101 in an A20 lymphoma model and demonstrated significant tumor regression and improved survival compared with vehicle and alum-alone controls (Figure 3D). We then tested mANK-101 in the nonimmunogenic B16F10 melanoma model and there was still a significant reduction in tumor growth and improved survival following 2 doses of mANK-101 (Figure 3E).

Alhydrogel is an established immune adjuvant, although its direct impact on immunity has been questioned. We did not see an impact of Alhydrogel alone when tested in the A20 tumor model (Figure 3D). To further rule out a major effect of intratumoral Alhydrogel alone on tumor outgrowth and antitumor immunity, we evaluated the impact of injecting vehicle, Alhydrogel, mIL12-ABP, or mANK-101 into established MC38 tumors. At 7 days, neither vehicle nor Alhydrogel significantly slowed tumor growth, whereas mIL12-ABP and mANK-101 slowed tumor growth at 7 days, with greater inhibition of tumor growth by mANK-101 than mIL12-ABP (Supplemental Figure 4A). Moreover, while neither vehicle nor Alhydrogel had an impact on plasma levels of IFN-γ, IL-12-ABP and mANK-101 led to a modest increase in plasma IFN-γ by 6 hours that increased at 2 days (Supplemental Figure 4B). At 7 days, the increase in plasma IFN-γ with IL-12-ABP returned to baseline, while the increase with mANK-101 persisted. Finally, we evaluated changes in CD8^+^ T cells by immunohistochemistry (IHC). Only i.t. mANK-101 was associated with a significant increase in intratumoral CD8^+^ T cells (Supplemental Figure 4, C and D).

The abscopal activity of i.t. mANK-101 monotherapy was assessed in C57BL/6 mice bearing dual-flank B16F10 tumors (Figure 3F). B16F10 tumors are highly refractory to immunotherapy, with no response to antibodies against either CTLA-4 or PD-1/PD-L1. Mice treated with 2 i.t. injections of mANK-101 in only the right flank tumor had significant delays in growth of both the injected and noninjected tumors, leading to an increase in median survival compared with vehicle from day 14 to day 31 (*P* < 0.001) (Figure 3F). The activity of i.t. mANK-101 was then assessed in the nonimmunogenic 4T-1 breast cancer model, which spontaneously develops lung metastases. A single i.t. injection of 5 μg mANK-101 delayed growth of the primary 4T1 tumor and decreased lung metastasis formation in 60% of animals (*P* < 0.001) (Figure 3G). Together, these data demonstrate that 1 or 2 i.t. injections of mANK-101 can induce regression of injected tumors across a range of established syngeneic tumors, promote regression of noninjected contralateral tumors, and may decrease metastasis, although we could not rule out the possibility that the reduction in metastatic lesions was related to smaller primary tumors. These findings together suggest that ANK-101 can induce both local and systemic immunity.

### Pharmacokinetic and pharmacodynamic assessment of ANK-101.

**Figure 4:**
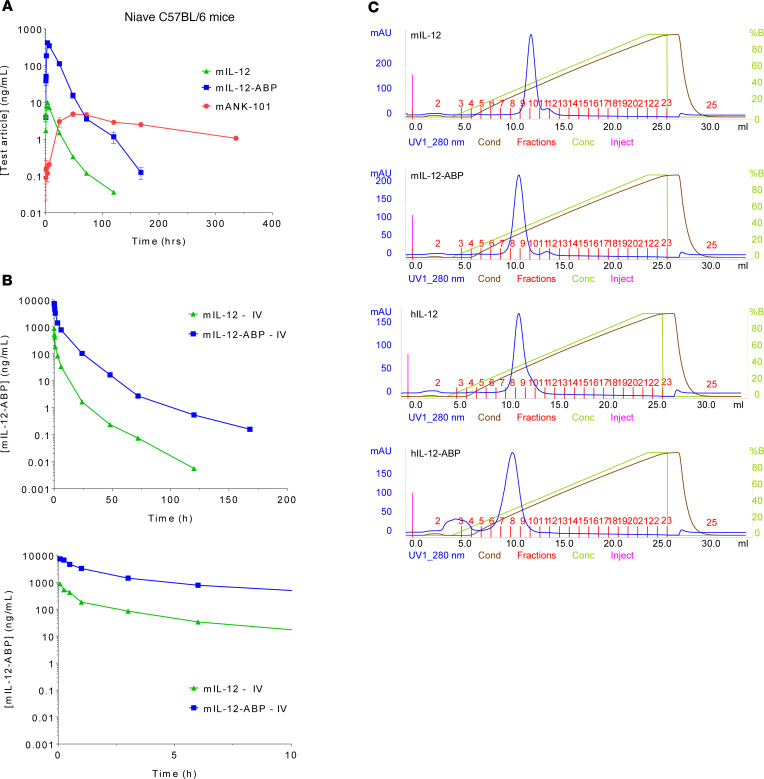
Pharmacokinetic and pharmacodynamic evaluation of mANK-101. (**A**) Drug concentration in serum following s.c. administration of unlabeled mIL-12, mIL-12-ABP, or mANK-101 in C57BL/6 mice as measured by murine IL-12-p70 MSD kit (*n* = 5, mean ± SEM). (**B**) Concentration of mIL-12 or mIL-12-ABP in serum following i.v. administration in C57BL/6 mice as measured by murine IL-12-p70 MSD kit showing full time course (top) or first 10 hours (bottom). (**C**) Elution of IL-12 or IL-12-ABP proteins from heparin HP HiTrap column during gradient from 0 to 1.5 M NaCl in 50 mM HEPES, pH 7.4.

We further characterized the mechanistic pharmacokinetics of mANK-101. Unlabeled mIL-12, free mIL-12-ABP, or mANK-101 was injected s.c. into C57BL/6 mice and plasma drug levels quantified over time with an IL-12p70 assay (Figure 4A). When free mIL-12-ABP protein was injected, mIl-12-ABP rapidly accumulated in the blood following s.c. administration, with a *C*_max_ of 418 ng/mL at 3 hours, followed by rapid clearance. In contrast, systemic accumulation of mIL-12-ABP protein given as mANK-101 complex was significantly reduced and delayed, with a *C*_max_ of 4.8 ng/mL at 48 hours. The terminal clearance rate of mANK-101 was also significantly slower, likely due to flip-flop kinetics where absorption from the injection site into the blood becomes rate limiting. It is interesting to note that wild-type mIL-12 without ABP had similar absorption and clearance kinetics as free mIL-12-ABP following either s.c. (Figure 4A) or i.v. (Figure 4B) administration, but a significantly lower amount of protein was detected. The low plasma mIL-12 levels may reflect sequestration on cells or tissues through heparin binding, as reported previously (42). We found, however, that both murine and human IL-12-ABP proteins have reduced affinity for heparin compared with the wild-type proteins, potentially due to electrostatic repulsion from the negatively charged ABP (Figure 4C and Supplemental Table 1)

**Figure 5:**
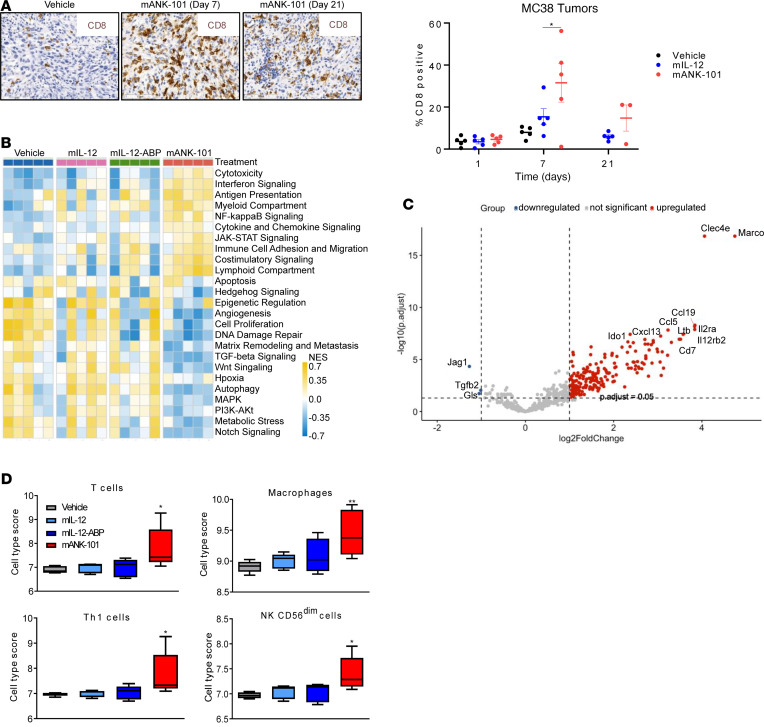
mANK-101 drives the i.t. accumulation of T cells, NK cells, and macrophages. (**A**) C57BL/6 mice (*n* = 5) bearing MC38 tumors were treated with a single i.t. injection of vehicle, 4.6 μg mIL-12, 5 μg free mIL-12-ABP protein, or 5 μg mANK-101, and CD8^+^ T cell infiltration by IHC was analyzed; representative images (left) and quantification of CD8^+^ T cells (right) are shown. (**B**) Mice bearing MC38 tumors were treated as in **A**, and i.t. gene expression was analyzed on day 7 by NanoString Mouse PanCancer IO360 panel. Differentially expressed genes between vehicle, mIL-12, mIL-12-ABP, and mANK-101 are shown in the heatmap representing changes in functional annotation pathways with treatment by normalized enrichment score (NES). (**C**) Volcano plot highlighting 203 genes’ expression following mANK-101 treatment with cutoff of *P*_adj_ < 0.05. (**D**) Cell type abundance with treatment as measured by average log-scale expression of gene expression signature. *P < 0.05, **P < 0.01, ***P < 0.0005 compared with vehicle by 1-way ANOVA with Dunnett’s post hoc test. IHC and FACS comparisons were performed by 1-way ANOVA with Tukey’s post hoc test.

With functional evidence of antitumor immunity induced by mANK-101, we interrogated the potential mechanisms by which mANK-101 could induce antitumor immunity using phenotypic analysis, bulk gene expression profiling of the TME, and single-cell transcriptomic analysis of the TME. IHC was performed to profile CD8^+^ T cells in MC38 tumors injected with vehicle, mIL-12, or mANK-101 (Figure 5A). ANK-101 injection increased CD8^+^ T cell infiltration that peaked around day 7 after injection, and then declined but persisted through day 21. In contrast, MC38 tumors treated with mIL-12 had a slight elevation of CD8^+^ T cells on day 7 that resolved to baseline by day 21; there was no change in CD8^+^ T cells in vehicle-treated tumors.

Next, C57BL/6 mice bearing MC38 tumors were given a single i.t. injection of vehicle, wild-type mIL-12, unanchored mIL-12-ABP protein, or mANK-101, and then sacrificed 6 hours or 7 days later. Treated tumors were excised and gene expression analyzed using the NanoString nCounter Mouse PanCancer IO360 Panel kit. Low levels of immune activation were observed at 6 hours in the mIL-12, mIL12ABP, and mANK-101 groups, with modest increases in IFN-γ, CXCL9, and CXCL10 (Supplemental Figure 5A). Significantly increased expression of CXCL3 and CXCL5 was also detected at 6 hours in the mANK-101–treated group only, potentially indicating alum-induced neutrophil recruitment as reported previously (42) (Supplemental Figure 5B).

On day 7 after injection, gene expression patterns in tumors from mice treated with mIL-12 or free mIL-12-ABP were somewhat similar to the gene expression patterns in vehicle-treated tumors (Supplemental Figure 5A). In contrast, mice treated with mANK-101 had robust gene changes on day 7, with 203 genes significantly upregulated compared with vehicle (Figure 5B). Upregulated genes included expected markers of IL-12 and IFN-γ signaling (IFN-γ, CXCL9, CXCL10, and IL-12-RB2), common γ chain cytokine receptors (IL-2RA and IL-7R), tertiary lymphoid structure signatures (CCL19, CXCL13, and Ltb), macrophage scavenger receptors (Clec4e and MARCO), and counterregulatory mechanisms known to be upregulated in response to IL-12 and IFN-γ (IDO and PD-L1) (Figure 5C). When analyzed by functional annotation pathways, mANK-101–treated tumors had significant upregulation of genes associated with immune cell activation and cytotoxicity, including IFN signaling, antigen presentation, and costimulatory signaling, and downregulation of pathways consistent with tumor growth and immune suppression, including TGF-β signaling and cell proliferation (Figure 5C). Gene expression signatures from mANK-101–treated tumors on day 7 were consistent with increased infiltration of T cells, Th1 cells, macrophages, and CD56^dim^ NK cells (Figure 5D). We also applied NanoString analysis of gene expression in MC38 tumors 7 days after treatment with vehicle, mIL-12-APB, alum alone, or mANK-101 and no gene expression changes were seen with Alhydrogel alone (Supplemental Figure 5C). These data suggest that there are significant gene expression changes in the TME mediated by the mIL-12 component of mANK-101.

**Figure 6:**
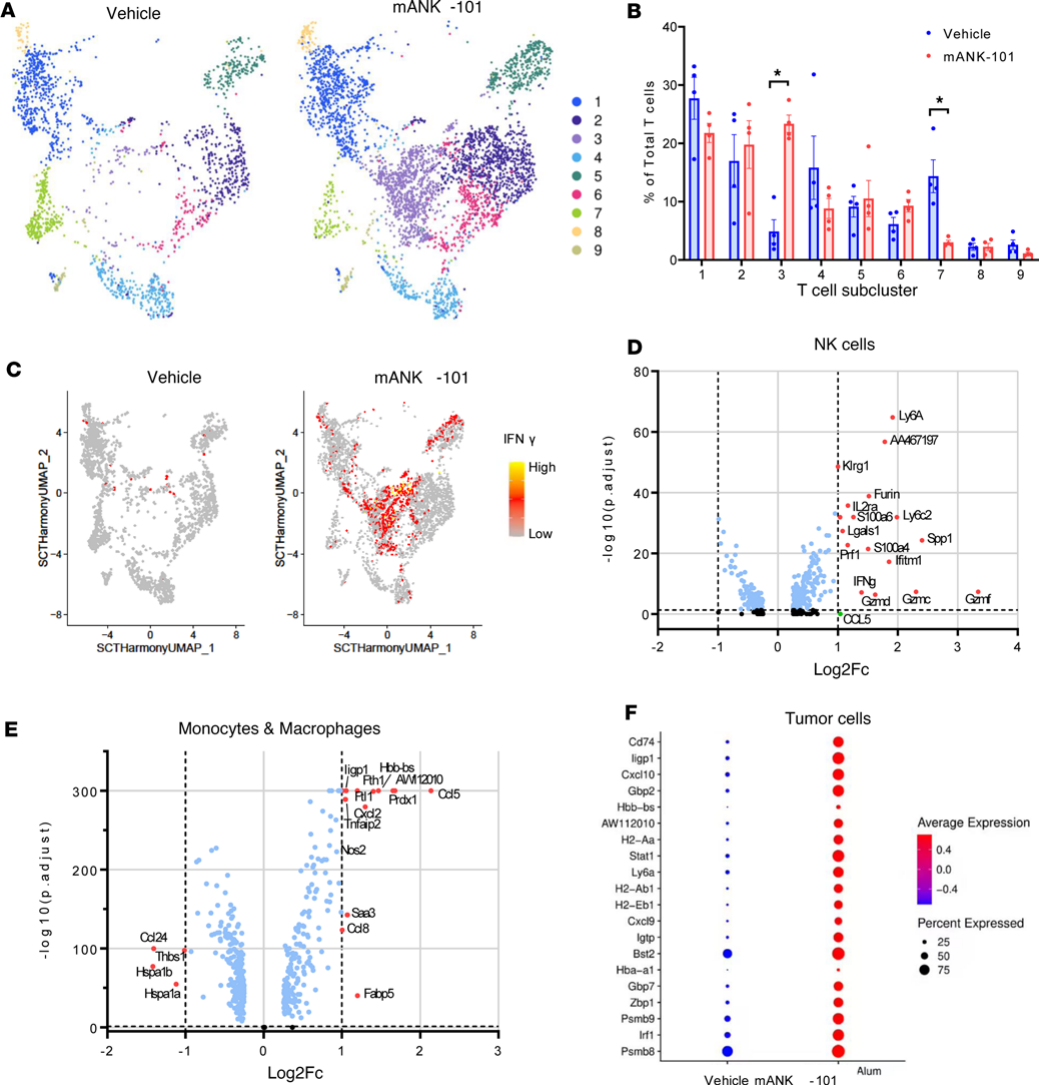
mANK-101 remodels the tumor microenvironment. C57BL/6 mice (*n* = 4) bearing MC38 tumors were treated with a single i.t. injection of vehicle or 5 μg mANK-101. On day 7, tumors were excised and analyzed by scRNA-seq. (**A**) UMAP plot of T cell subsets including 9 spiking neural network clusters found using a resolution parameter of 0.4. (**B**) Changes in relative proportion of T cell subclusters between vehicle- and mANK-101–treated animals. *P < 0.05 by Mann-Whitney test. (**C**) Relative IFN-γ expression overlaid on T cell UMAP from vehicle- or mANK-101–treated mice. (**D** and **E**) Differentially expressed transcripts between vehicle- and mANK-101–treated mice within NK cell and monocyte and macrophage cell clusters. Red dots represent transcripts that were increased or decreased at least 2-fold after treatment, with *P*_adj_ < 0.05. Log2Fc, log_2_(fold change). (**F**) Bubble plot showing the top 20 differentially expressed transcripts within the tumor cell cluster between vehicle- and mANK-101–treated animals. Dot color represents average relative expression of each transcript, while dot size indicates the percentage of cells expressing the transcript above background.

To obtain a more comprehensive understanding of i.t. immune changes, single-cell transcriptomic analysis was performed on MC38 tumors 7 days after a single i.t. injection of vehicle or mANK-101. Single-cell clustering by gene expression analysis identified distinct clusters of cancer, stromal, lymphoid, and myeloid cells (Supplemental Figure 6A). mANK-101–treated animals had 2-fold higher T cells on average than vehicle-treated animals (Supplemental Figure 6B). Further subclustering of T cells from vehicle- and mANK-101–treated animals identified 9 subclusters (Figure 6A and Supplemental Figure 6C). Compared with control animals, mANK-101–treated mice had significantly reduced cells in cluster 7, identified as Tregs (*Foxp3* and *Ctla4*), and significantly higher cells in cluster 3, consisting primarily of CD4^+^ T cells expressing high levels of IFN-γ (Figure 6, B and C). Similar Th1-like CD4^+^ populations have been reported in mouse tumors after IL-12 or anti-CD40 treatment and may represent conversion of fragile Tregs to a more proinflammatory phenotype (43, 44). Tumors from mANK-101–treated animals also had more cells in cluster 2 (*Cd8*, *Klrd1*, *Nkg7*, and *Gzmf*) and cluster 6 (*Cd8*, *Gzma*, *Gzmb*, *Prf1*, and *Ccl5*), reflecting 2 populations of cytotoxic effector T cell populations, with a trend toward high numbers of cycling T cells (*Mki67*) in cluster 5 (Figure 6B).

mANK-101 treatment led to activation of intratumoral NK cells, with increased expression of *Ifng*, *Ly6a*, *Klrg1*, *Prf1*, and *Gzmc/d/f*, reflecting direct IL-12 signaling on NK cells (Figure 6D). mANK-101 treatment also induced changes in monocyte and macrophage populations, with significantly increased expression of chemokines *Ccl5*, *Cxcl2*, and *Ccl8* and decreased expression of *Ccl24*, as well as increased expression of the M1 marker *Nos2* (Figure 6E). Tumor cell gene expression also changed significantly in mANK-101–treated animals, with increased *Stat1*, *Cxcl9*, and *Cxl10* (all consistent with IFN-γ signaling), as well as increases in *Cd74*, MHC alleles, and proteasome components, indicating increased antigen processing and presentation (Figure 6F). Together, these data indicate that a single i.t. injection of mANK-101 induces a dramatic and sustained remodeling of the TME, with increased recruitment of T cells via a chemokine gradient, a Th1 phenotype shift, decreased Tregs, increased CD8^+^ T cell and NK cell activation and cytotoxicity, myeloid cell skewing to a proinflammatory M1 phenotype, and enhanced antigen processing and presentation by tumor cells.

### mANK-101 administration synergizes with systemic immune checkpoint blockade.

**Figure 7:**
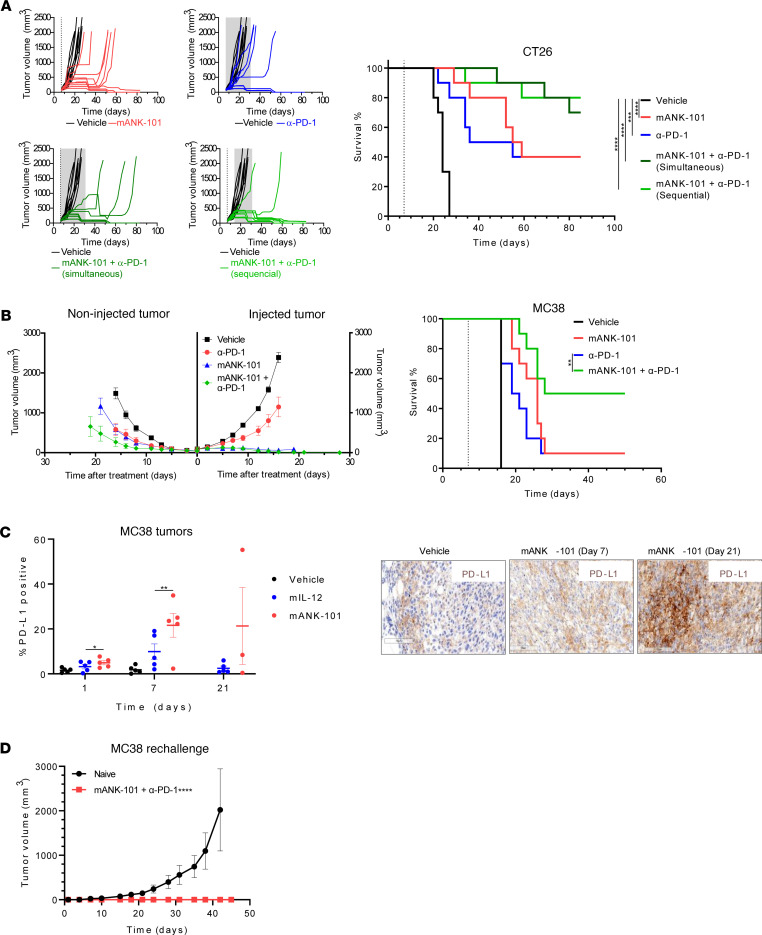
mANK-101 improves therapeutic responses in combination with PD-1 blockade. (**A**) Tumor growth (left) and survival (right) of mice (*n* = 10) bearing CT26 tumors treated with a single i.t. injection of vehicle or 5 μg mANK-101 alone or in combination with anti–mouse PD-1 biweekly for up to 4 weeks. Anti–PD-1 treatment started on the same day as mANK-101 administration (simultaneous) or 1 week later (sequential). The dashed vertical line denotes the mANK-101 treatment day and shaded gray area represents the duration of anti–PD-1 treatment. (**B**) Tumor growth (left) and survival (right) for mice bearing dual-flank MC38 tumors treated with a single i.t. injection of vehicle or 5 μg mANK-101 in the right flank tumor only, alone or in combination with anti–mouse PD-1 antibody dosed 3 mg/kg i.p. biweekly. (**C**) Mice (*n* = 10) bearing MC38 tumors were treated with a single i.t. injection of vehicle, 4.6 μg mIL-12, or 5 μg mANK-101 and tumors analyzed by IHC for PD-L1 expression over time; quantification (right) and representative images from 1 mouse (left) are shown. (**D**) Tumor growth of mice (*n* = 10) bearing MC38 tumors that had complete response to mANK-101 and anti–PD-1 were rechallenged 100 days later with MC38 tumors. Controls were aged-matched naive mice challenged with MC38. *P < 0.05; **P < 0.01; ***P < 0.0005; ****P < 0.0001 by log-rank Mantel-Cox test (survival curves in **A** and **B**) or 1-way ANOVA with Tukey’s post hoc test (**C** and **D**).

Since mANK-101 increases expression of PD-L1, which can suppress antitumor immunity, we evaluated the combination of mANK-101 with either PD-1 or CTLA-4 blockade in BALB/c mice bearing CT26 tumors. Tumor-bearing mice were given a single i.t. injection of mANK-101 alone or combined with PD-1 or CTLA-4 blockade starting either simultaneously (concurrent) or 1 week after (sequential) mANK-101 injection. While CT26 tumor–bearing animals treated with mANK-101 or systemic PD-1 blockade alone had a 50% tumor-free survival rate, the combination was associated with tumor-free survival rates of 70% and 80% for the concurrent and sequential dosing groups, respectively (Figure 7A). Similarly, CT26 tumor–bearing animals treated with mANK-101 or systemic CTLA-4 blockade alone had 40% and 60% tumor-free survival rates respectively, while the combination had a 90% tumor-free survival rate (Supplemental Figure 7). We also characterized the combination of mANK-101 and anti–PD-1 in the MC38 model. MC38 tumor–bearing animals treated with mANK-101 or systemic PD-1 blockade alone each had a tumor-free survival rate of 40%, whereas the combination of ANK-101 and CTLA-4 blockade had tumor-free survival rates of 70% and 80% with concurrent or sequential therapy, respectively (data not shown). We also assessed the impact of mANK-101 combined with systemic PD-1 blockade on noninjected lesions. C57BL/6 mice bearing dual-flank MC38 tumors were treated with a single i.t. injection of mANK-101 in only the right flank tumor either alone or in combination with systemic anti–PD-1 (Figure 7B). Anti–PD-1 monotherapy delayed tumor growth in both flanks, resulting in only 10% tumor-free survival. A single mANK-101 injection induced complete regression in 90% of injected tumors but was only partially efficacious against noninjected lesions, delaying their growth in all mice and producing 10% tumor-free survival. The combination of a single i.t. injection of mANK-101 with systemic PD-1 blockade demonstrated optimal efficacy, with clearance of 100% of injected tumors and 50% of noninjected tumors, leading to a tumor-free survival rate of 50%.

High levels of IFN-γ are known to increase PD-L1 expression as a mechanism of adaptive resistance to prevent excessive immune activation (45). We evaluated levels of PD-L1 expression by IHC in MC38 tumors treated with a single injection of vehicle, mIL-12 alone, or mANK-101 on days 1, 7, and 21 after injection. Vehicle alone induced no significant changes. mIL-12 alone induced PD-L1 expression by 7 days, which returned to baseline by day 21. In contrast, mANK-101 alone induced higher levels of PD-L1 than mIL-12 alone at 7 days, and PD-L1 levels remained elevated on day 21 (Figure 7C). MC38-bearing mice achieving complete responses to mANK-101 and PD-1 blockade were rechallenged with MC38 tumors 100 days later and all mice were protected from tumor growth, suggesting durable memory responses (Figure 7D). Together, these data suggest that a single i.t. injection of mANK-101 can prime an antitumor response that is maintained and enhanced by systemic PD-1 blockade, with development of long-term tumor-specific memory.

### Subcutaneous administration of ANK-101 is well tolerated in cynomolgus monkeys.

We did not observe any systemic signs of toxicity or weight changes in any of the tumor models (Supplemental Figure 8A). Since ANK-101 is not cross-reactive in rodents, an exploratory toxicology study was performed in cynomolgus macaques, which have 94% and 96% sequence homology with humans for IL-12α and IL-12β, respectively. ANK-101 was administered s.c. to model local retention and systemic leakage, with 2 animals per group given a single injection of 0.2, 2, or 20 μg/kg ANK-101, followed by 2 animals per group receiving 2 repeat s.c. injections 7 days apart at the 2 or 20 μg/kg dose level. Previous studies in nonhuman primates with wild-type human IL-12 administered i.v. or s.c. reported that doses above 1 μg/kg can induce toxicity, including anorexia, diarrhea, fatigue, and colitis, in line with toxicity associated with the maximum tolerated dose of 0.3–1 μg/kg for IL-12 defined in human clinical studies (8).

**Figure 8:**
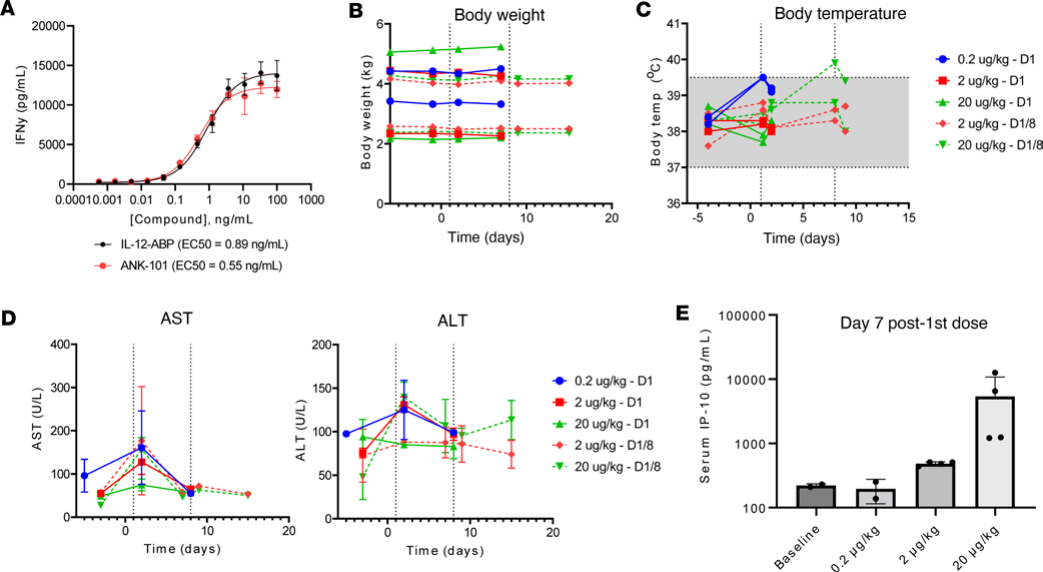
ANK-101 is well tolerated in nonhuman primates. (**A**) IFN-γ production measured by MSD assay from activated cynomolgus macaque PBMCs following 3-day incubation with a titration of free IL-12-ABP protein or ANK-101 complex (mean ± SD). (**B**–**D**) Cynomolgus macaques (*n* = 2 per group) were administered a single or repeat s.c. injection of mANK-101 at 0.2, 2, or 20 μg/kg and analyzed over time for changes in (**B**) body weight and (**C**) body temperature, (**D**) serum concentrations of aspartate transaminase(AST) and alanine transaminase (ALT), and (**E**) serum IP-10 was measured by MSD assay (mean ± SD).

Free human IL-12-ABP protein and ANK-101 both induced IFN-γ expression from activated cynomolgus macaque PBMCs in vitro, confirming biological cross-reactivity (Figure 8A). ANK-101 was well tolerated in all groups, with no treatment-related clinical observations, no injection site reactions, no change in body weight (Figure 8B), temperature (Figure 8C), coagulation (Supplemental Figure 8B), or hematology (Supplemental Figure 8C) parameters, and no macroscopic organ observations (data not shown). A subset of animals had mild, transient increases in aspartate transaminase 1 day after the first dose that fully resolved by day 7 (Figure 8D). There was no clear dose dependence to this response, and further increases were not observed after the second dose. Other clinical chemistry markers were unchanged (Supplemental Figure 8D). Animals in the high-dose group had elevations in systemic CXCL10 (IP-10) on day 7 after the first dose, confirming biological activity of ANK-101 (Figure 8E). Two of the animals also had increases in systemic IFN-γ (Supplemental Table 2). Serum concentrations of IL-1β, IL-2, IL-6, IL-10, and TNF-α were unchanged. These data support the systemic safety profile of ANK-101.

## Discussion

Following approval of the first i.t. administered oncolytic virus, talimogene laherparepvec for treatment of metastatic melanoma in 2015, there has been increased interest in approaches to locally deliver agents directly to the tumor, thereby increasing efficacy and reducing systemic toxicity. Local delivery may also better match the natural mechanism of immune activation for cytokines like IL-12, which evolved to drive a local immune reaction at sites of active infection or trauma (4). Despite these advances, the potential therapeutic benefit of i.t. administration remains limited by rapid clearance of injected drug from the tumor, thereby reducing local immune stimulation or requiring frequent injections, as well as increasing systemic exposure. This can be especially problematic for less accessible tumors where only one or a small number of injections may be technically feasible.

Multiple approaches have been recently reported to increase tumor retention after local administration. Protein modifications such as PEGylation, fusion to bulky peptide domains, or immobilization on liposomes or exosomes can increase molecular size within the nanometer range, leading to slower diffusion and release from the tumor. However, the increased retention of molecules in this size range is often modest, with the majority of drug still released within 1 or 2 days (28, 46). Immobilization in biodegradable matrices can further enhance retention but typically inhibits cytokine activity until the protein is released (47, 48). If the release kinetics are not well matched to cellular consumption, there may be insufficient activation or excessive release into circulation (34). Binding to tumor antigens or ECM components in the tumor can enhance retention but may be limited by heterogeneous or insufficient antigen levels in patient tumors (22). Similarly, local cytokine expression from i.t. administered mRNA, DNA, or oncolytic virus has demonstrated encouraging response rates in preclinical and early-phase clinical studies, but expression levels can vary significantly across patients or tumors, leading to dosing complexity and the potential for significant systemic leakage of unanchored cytokine (30–32).

Various immobilization strategies have been tested. One evaluated the delivery of cytokines fused to the collagen-binding protein lumican in multiple tumor-bearing mouse models, showing prolonged local retention, reduced systemic toxicity, and decreased tumor growth of the injected lesion with an abscopal effect. This approach was evaluated in a canine clinical trial that showed reprogramming of the TME in canine soft tissue sarcomas by IL-2 and IL-12 linked to lumican. Furthermore, in a prior report, local delivery and retention of IL-12 tethered to the FDA-approved vaccine adjuvant Alhydrogel demonstrated profound CD8^+^ T cell–dependent therapeutic activity compared with unanchored cytokine injections. Due to the large size (1–10 μm) and charge of Alhydrogel particles, complexed drug is retained in the tumor for a significantly longer duration than competing technologies based on size or antigen binding. In contrast to other biomaterial approaches, the cytokine remains active while bound, avoiding issues of matching release kinetics with consumption. Additionally, it is a tumor-agnostic approach in which the concentration and retention of active drug does not depend on any tumor-intrinsic property such as protein expression, protease concentration, or target antigen concentration.

Here, we move this technology toward clinical translation with characterization of ANK-101, composed of human or murine IL-12 fused to an optimized alum-binding peptide (IL-12-ABP) complexed with a 10-fold mass excess of Alhydrogel. By engineering the ABP sequence motifs and coexpressing under optimized Fam20C conditions, multiple phosphates can be reproducibly added to the IL-12-ABP proteins, with a mean of 8 and 6 phosphates for human and murine mIL-12-ABP, respectively. These phosphoserines mediate high-affinity binding to Alhydrogel even in the presence of phosphate and serum where wild-type IL-12 proteins are rapidly eluted. Free IL-12-ABP and ANK-101 induce IFN-γ expression from human PBMCs, CD8^+^ T cells, and NK cells with similar potency.

Following i.t. administration in a syngeneic CT26 tumor, mANK-101 is retained in the TME, with 31%–37% of the injected dose remaining on day 21 as quantified by ^125^I SPECT/CT imaging, and i.t. protein detectable on day 28 by IVIS imaging. In contrast, free mIL-12-ABP protein delivered i.t. is rapidly cleared from the tumor, with approximately 3% remaining at 24 hours. The enhanced local retention of mANK-101 also reduces systemic exposure of mIL-12-ABP, with a 14- to 72-fold decrease in serum *C*_max_ compared with free IL-12-ABP delivered i.t., depending on the model.

mANK-101 delivered i.t. is highly efficacious as a single agent in diverse syngeneic tumor models, ranging from immunogenic tumors across different genetic strains, such as CT26 and MC38, to nonimmunogenic “cold” tumors, such as B16F10 and 4T-1 that do not respond to most IO agents, including immune checkpoint blockade. While mANK-101 demonstrated significant tumor growth inhibition and improved survival across all these models, wild-type mIL-12 and free mIL-12-ABP protein had no efficacy compared to vehicle when administered at an identical dose and frequency as mANK-101. Injection of mANK-101 i.t. into the primary tumor both reduced formation of spontaneous lung metastases in an orthotopic 4T-1 model and led to delays or regressions of noninjected tumors in dual-flank MC38 or B16F10 models, especially when combined with systemic checkpoint blockade. This supports induction of systemic antitumor immunity, although we could not completely exclude that the impact on pulmonary metastasis may have been due to treatment of the primary tumor. Nonetheless, tumor rechallenge experiments 100 days after treatment demonstrated protection against a subsequent tumor challenge, suggesting durable tumor-specific memory responses could be generated by mANK-101 treatment. One or 2 injections of mANK-101 was sufficient in most models, suggesting that this approach may be ideal for both the treatment of less accessible lesions requiring more invasive procedures, and for neoadjuvant therapy.

mANK-101 treatment was associated with enhanced infiltration of T cells, along with a shift to a Th1 phenotype with increased levels of IFN-γ–expressing CD4^+^ T cells and cytotoxic effector CD8^+^ T cells. Beyond T cells, there was evidence of increased NK cell activation, with upregulation of IFN-γ and cytotoxic markers, including perforin and granzyme, likely due to direct IL-12 signaling. Similarly, mANK-101 treatment induced myeloid cell activation, with increased markers of M1 skewing, costimulation, and antigen presentation, including iNOS, CD86, MHCII, and CCL5. Tumor cells also underwent significant changes after mANK-101 treatment, including increased expression of proinflammatory chemokines CXCL9 and CXCL10, and upregulation of genes responsible for antigen processing and MHC I presentation, likely mediated by IFN-γ signaling through STAT1. Importantly, there was no significant remodeling of the TME with i.t. administration of Alhydrogel or free IL-12-ABP alone (Supplemental Figure 5C). Together, these pleiotropic immune changes suggest that IL-12 sustainably delivered as mANK-101 may convert immunologically “cold” tumors to a more immune-responsive phenotype.

mANK-101 was well tolerated in murine efficacy studies, with no measurable body weight loss at efficacious doses. In an exploratory toxicology study in cynomolgus macaques with s.c. administration of ANK-101 at doses exceeding the previous human MTD for wild-type IL-12, ANK-101 was well tolerated in all groups, with no treatment-related clinical observations, or change in body weight, temperature, coagulation or hematology measurements, and no macroscopic organ observations. Together, these data demonstrate that IL-12 anchored to Alhydrogel may represent an approach to cancer immunotherapy with a more favorable treatment schedule and improved therapeutic window. Based on these promising preclinical findings, ANK-101 is progressing toward clinical studies in patients with solid tumors.

## Methods

Further details can be found in the Supplemental Methods.

### Malachite green assay.

Phosphorylation of purified proteins was measured using the Pierce Phosphoprotein Phosphate Estimation Assay Kit (Thermo Fisher Scientific) per manufacturer’s instructions.

### Alum retention assay.

Human or mouse IL-12-ABP protein (0.25 mg/mL) was mixed with 2.5 mg/mL Alhydrogel in TBS and incubated for 30 minutes to form ANK-101 or mANK-101 complexes. Complexes were incubated at 37°C in TBS with or without 40% serum and 1 mM phosphate. At various times, samples were centrifuged to pellet the Alhydrogel and free IL-12-ABP was measured in the supernatant using an ELISA against mouse IL-12 (R&D Systems, m1270) or human IL-12 (R&D Systems, 24910 for capture; BioLegend, 508801 for detection).

### Murine syngeneic early intervention tumor efficacy models.

CT26, B16F10, and 4T-1 cells were obtained from Shanghai Institutes for Biological Sciences. A20 cells were purchased from ATCC and MC38 cells were obtained from the National Cancer Institute/NIH. All cells were maintained at 37°C and 5% CO_2_ in RMPI 1640 with 10% FBS for CT26 and 4T-1, DMEM plus 10% FBS for B16F10, RPMI 1640 plus 10% FBS and 0.05 mM β-ME for A20, and DMEM plus 20 mM glutamine and 10% FBS for MC38. We utilized an early intervention model in which female BALB/c mice aged 7–9 weeks or C57BL/6 mice aged 6–8 weeks (Beijing Vital River Laboratory Animal Technology Co, Ltd.) were inoculated s.c. on the right flank with 5 × 10^5^ tumor cells for CT26 and A20 models or 1 × 10^6^ tumor cells for B16F10 and MC38 models. For dual-flank models, the same number of cells was inoculated s.c. in the left flank 3 days (B16F10) or 2 days (MC38) after inoculation of the right-flank tumor. For the orthotopic 4T-1 model, 3 × 10^5^ 4T-1 cells were injected into the right mammary fat pad. Unless otherwise specified, mice were treated when mean tumor volume reached 70–110 mm^3^. Intratumoral injections were performed in a 20 μL injection volume using TBS as the vehicle. For mice treated with mANK-101 complex, 0.25 mg/mL mIL-12-ABP was mixed with 2.5 mg/mL Alhydrogel and incubated for 30 minutes to form a complex prior to injection. Tumor growth was monitored by caliper measurements and tumor volume (TV) calculated as TV = (L × W^2^)/2, where L is tumor length (longest dimension) and W is tumor width (longest dimension perpendicular to L). Mice were euthanized when tumor area exceeded 2000 mm^3^ or in the case of significant tumor ulceration or weight loss beyond 20%. An independent experiment was performed with an alum-alone control in the A20 and MC38 tumor models.

### IVIS.

mIL-12-ABP protein was covalently labeled on primary amines with NHS–Alexa Fluor 647 (A647) fluorophore (Thermo Fisher Scientific). BALB/c mice bearing approximately 100-mm^3^ tumors were treated with a single i.t. injection of vehicle, 5 μg free A647-IL-12-ABP, or 5 μg A647-IL-12-ABP complexed with 50 μg Alhydrogel. Whole-body fluorescence images were obtained at various times using an IVIS Lumina Series III and analyzed using Living Image 4.5 software (PerkinElmer).

### Radiolabeled biodistribution studies.

mIL-12-ABP protein was covalently labeled on primary amines with ^125^I radioisotope (PerkinElmer) using succinimidyl iodobenzoate (SIB) chemistry to a specific activity of 9.0 μCi/μg and then adjusted to 5.0 μCi/μg by adding cold test article. BALB/c mice bearing CT26 tumors (ATCC) received a single i.t. injection targeting 5 μg (25 μCi) of ^125^I-mIL-12-ABP either as free protein or complexed with Alhydrogel to form mANK-101 in a 20 μL injection volume. The dosing syringe was assayed in a dose calibrator before and after injection to determine the actual activity administered to each animal. Whole-body SPECT/CT images were acquired at multiple time points on a NanoScan device (Mediso) with acquisition and reconstruction parameters listed in Supplemental Table 3. Gamma counts per minute (CMP) were measured from serial whole-blood samples collected from the submandibular vein and tissues collected from individual mice sacrificed between 72 and 504 hours. Values were decay corrected to the time of injection and corrected for background radiation. Additional details on the ^125^I-SIB labeling and SPECT/CT image analysis are included in the Supplemental Methods.

### Cytokine analysis.

For i.t. IFN-γ measurements, snap-frozen B16F10 tumors were mixed twice with ice-cold cell lysis buffer with protease/phosphatase inhibitor cocktail (Cell Signaling Technology) and homogenized using a Tissue Lyser II (Qiagen) at 30 Hz for 10 minutes. Samples were incubated at room temperature for 30 minutes with gentle shaking and then centrifuged and supernatants collected. Total protein was quantified by BCA assay (Pierce) and IFN-γ measured using a V-PLEX proinflammatory panel mouse kit (Meso Scale Discovery [MSD], K15048D). Systemic drug levels were measured in plasma using an MSD V-PLEX mouse IL-12p70 kit (K152QVD-2) with mIL-12 or mIL-12-ABP used for the standard curves.

### In vitro immune assays.

Human PBMCs from healthy volunteer donors were isolated from commercially sourced buffy coats by Ficoll centrifugation gradient (GE Healthcare Ficoll-Paque PLUS Media). Purified CD8^+^ T cells and NK cells were isolated by magnetic separation. PBMCs (5 × 10^5^), CD8^+^ T cells (2 × 10^5^), or NK cells (1.5 × 10^4^) were seeded in round-bottom 96-well plates. Cells were treated with a titration of IL-12, free IL-12-ABP, or ANK-101 complex in the presence of 100 ng/mL anti-CD3 antibody (clone OKT3, eBioscience) for PBMCs and T cells or 10 ng/mL IL-1β for NK cells. After 3 days, supernatants were harvested and IFN-γ concentration measured by time-resolved fluorescence resonance energy transfer (TR-FRET). Purified cynomolgus macaque PBMCs were purchased from BioIVT and analyzed as above using a nonhuman primate–specific CD3 antibody (Mabtech, 3610-1-50) for stimulation and measuring IFN-γ by MSD assay (kit K156QOD).

### Flow cytometry.

Tumor tissues, inguinal lymph nodes from the same flank, spleens, and whole blood were collected at study termination. Tissues were dissociated to single cells using a Miltenyi Biotec GentleMACS for tumor and spleen or manual pushing through a 70-μm strainer for lymph nodes. Cells were Fc-blocked using Mouse BD Fc Block (BD Biosciences) and spleen and whole blood samples were treated with red blood cell lysing buffer (BioGems). Cells were stained with the panel of antibodies listed in Supplemental Table 4 in Fc-block buffer, and then permeabilized using a Foxp3 Fix/Perm kit (eBioscience) and stained for Foxp3. For cell counting, 123count eBeads (eBioscience) were added to each sample prior to analysis. Stained cells were analyzed on an LSRFortessa X-20 (BD Biosciences) and the data were analyzed with Kaluza software (Beckman Coulter).

### NanoString.

RNA was extracted from syngeneic tumor tissues frozen in RNAlater (Qiagen) using an RNeasy Mini Kit (Qiagen). RNA (100–300 ng) was evaluated using the nCounter Mouse PanCancer IO360 Panel (NanoString Technologies). Gene counts were normalized based on housekeeping genes using the median-of-ratio method. Differentially expressed gene analysis was performed using Wald’s test, and *P* values were corrected for multiple testing with the Benjamini-Hochberg method. Significant genes were defined as an adjusted *P* value (*P*_adj_) of less than 0.05 and fold change greater than 2. Functional enrichment analysis was based on gene set variation analysis, and statistical tests were performed using the limma package (https://bioinf.wehi.edu.au/limma/) based on empirical Bayes shrinkage method. Gene signatures used in cell type and pathway analyses are listed in Supplemental Table 5.

### IHC.

Syngeneic tumor tissues were fixed in 10% formalin, embedded in paraffin, and sectioned at a 4 μm thickness on a Leica RM2235 microtome. Sections were stained with rabbit monoclonal antibodies (Cell Signaling Technology) against murine CD8α (clone D4W2Z) and PD-L1 (clone D5V3B) using the automated Leica BOND Rx platform. Stained slides were imaged with a Leica Aperio VERSA scanner and analyzed with HALO software (Indica Labs). The percentage positivity for each marker was calculated as the density of positive cells per mm² divided by the total number of cells per mm².

### Single-cell RNA sequencing.

MC38 tumors were dissociated to single cells, subjected to RBC lysis, washed twice with Dulbecco’s PBS and 1% BSA, and normalized to approximately 1000 cells/μL. Cells were loaded onto a 10× Genomics Chip G Single Cell kit, single-cell capture was performed, and libraries were prepared per manufacturer’s instructions. Samples were 150-bp, paired-end sequenced on the Illumina NovaSeq 6000 platform, targeting a median sequencing depth of 50,000 read pairs/cell for each sample. The 10× Cell Ranger Software Suite (v5.0.1) was used to perform sample demultiplexing, barcode processing, and single-cell 3′ UMI counting with mouse GRCm38 as the reference genome. Following manual annotation, cells were divided into tumor cells, T cells, monocyte/macrophages, or NK cell subgroups for subsequent analyses of these individual cell types. The subsets of cells were renormalized using SCTransform (https://github.com/satijalab/sctransform) followed by PCA and UMAP dimension reduction. Unsupervised cluster identification (spiking neural network) was performed using FindClusters. Transcripts most differentially expressed between vehicle- and mANK-101–treated mice or between cell clusters was performed using FindMarkers (binary contrasts) or FindAllMarkers (multiple contrasts) algorithms and Wilcoxon’s rank-sum test, and *P* values adjusted with Bonferroni’s correction. All analyses were done using Seurat (v4.1.0) software (https://satijalab.org/seurat) and default parameters unless otherwise indicated. The data discussed in this publication have been deposited in NCBI’s Gene Expression Omnibus and are accessible through GEO Series accession number GSE245465.

### Nonhuman primate studies.

Naive cynomolgus macaques, age 2.5–5 years, were administered a single s.c. injection of 0.2, 2, or 20 μg/kg ANK-101 on day 1 or 2 repeat administrations of 2 or 20 μg/kg ANK-101 on days 1 and 8 with 2 animals (1/sex) per group. Clinical observations, injection site observations, body weights, and body temperatures were recorded. Whole blood or serum samples were collected at various times and analyzed for hematology parameters (Supplemental Table 6) on an ADVIA 2120 system, clinical chemistry parameters (Supplemental Table 7) using a TBA-120FR analyzer, coagulation parameters using a Sysmex analyzer, and systemic concentrations of IL-2, IL-6, IL-10, TNF-α, IL-1β, IL-12p70, IFN-γ, and CXCL10 using MSD kit K15068L.

### Statistics.

Statistical analyses were performed with GraphPad Prism 9.0 software. Survival in all the efficacy studies utilized the Kaplan-Meier method and statistical significance compared to the vehicle treatment group was calculated by log-rank Mantel-Cox test. IHC and FACS comparisons were performed by 1-way ANOVA with Tukey’s post hoc test. For the NanoString analyses, comparison to vehicle performed by 1-way ANOVA with Dunnett’s post hoc test. All values are expressed as the mean ± SEM unless otherwise noted. In all analyses, statistical significance was set at *P* less than 0.05.

### Study approval.

All procedures involving the care and use of animals in the studies were performed according to the guidelines approved by the respective Institutional Animal Care and Use Committees of Crown Bio, Wuxi AppTec, InVicro, Inc., and JOINN Laboratories, Inc, following the guidance of the Association for Assessment and Accreditation of Laboratory Animal Care (AAALAC).

### Data and materials availability.

scRNA-seq data are deposited in the NCBI GEO (GSE245465). All other data are available in the main text or the supplemental materials. A Supporting Data Values Excel file, including all the raw data for Figures 1–8, is provided in the supplemental material.

## Author contributions

SB, KDW, and MMS conceptualized the study. SB, GP, and MMS developed the methodology. SB and MMS carried out the investigation. SB and MMS generated figures. KDW, HLK, and MMS supervised the study. SB and MMS wrote the original draft of the manuscript, which was reviewed and edited by SB, HLK, KDW, and MMS.

## Supplementary Material

undefined

Supplemental data

Supporting data values

## Version 1 12/08/2023

Electronic publication

## References

[1] Zou W (2016). PD-L1 (B7-H1) and PD-1 pathway blockade for cancer therapy: mechanisms, response biomarkers, and combinations. Sci Transl Med.

[2] Briukhovetska D (2021). Interleukins in cancer: from biology to therapy. Nat Rev Cancer.

[3] Holder PG (2022). Engineering interferons and interleukins for cancer immunotherapy. Adv Drug Deliv Rev.

[4] Liu J (2005). Interleukin-12: an update on its immunological activities, signaling and regulation of gene expression. Curr Immunol Rev.

[5] Lasek W (2014). Interleukin 12: still a promising candidate for tumor immunotherapy?. Cancer Immunol Immunother.

[6] Brunda MJ (1993). Antitumor and antimetastatic activity of interleukin 12 against murine tumors. J Exp Med.

[7] Brunda MJ (1996). Antitumor activity of interleukin 12 in preclinical models. Cancer Chemother Pharmacol.

[8] Leonard JP (1997). Effects of single-dose interleukin-12 exposure on interleukin-12-associated toxicity and interferon-gamma production. Blood.

[9] Car BD (1999). The toxicology of interleukin-12: a review. Toxicol Pathol.

[10] Cohen J (1995). IL-12 deaths: explanation and a puzzle. Science.

[11] Atkins MB (1997). Phase I evaluation of intravenous recombinant human interleukin 12 in patients with advanced malignancies. Clin Cancer Res.

[12] Portielje JE (1999). Phase I study of subcutaneously administered recombinant human interleukin 12 in patients with advanced renal cell cancer. Clin Cancer Res.

[13] Aznar MA (2017). Intratumoral delivery of immunotherapy—act locally, think globally. J Immunol.

[14] Melero I (2021). Intratumoural administration and tumour tissue targeting of cancer immunotherapies. Nat Rev Clin Oncol.

[15] Yuan J (2021). Current strategies for intratumoural immunotherapy – beyond immune checkpoint inhibition. Eur J Cancer.

[16] Nguyen KG (2020). Localized interleukin-12 for cancer immunotherapy. Front Immunol.

[17] Hong WX (2020). Intratumoral immunotherapy for early-stage solid tumors. Clin Cancer Res.

[18] Brody JD (2010). In situ vaccination with a TLR9 agonist induces systemic lymphoma regression: a phase I/II study. J Clin Oncol.

[19] Danielli R (2015). Intralesional administration of L19-IL2/L19-TNF in stage III or stage IVM1a melanoma patients: results of a phase II study. Cancer Immunol Immunother.

[20] Algazi A (2020). Intratumoral delivery of tavokinogene telseplasmid yields systemic immune responses in metastatic melanoma patients. Ann Oncol.

[21] Chesney J (2017). Randomized, open-label phase II study evaluating the efficacy and safety of talimogene laherparepvec in combination with ipilimumab versus ipilimumab alone in patients with advanced, unresectable melanoma. J Clin Oncol.

[22] Momin N (2019). Anchoring of intratumorally administered cytokines to collagen safely potentiates systemic cancer immunotherapy. Sci Transl Med.

[23] Kwong B (2011). Induction of potent anti-tumor responses while eliminating systemic side effects via liposome-anchored combinatorial immunotherapy. Biomaterials.

[24] Van Herpen CM (2004). Intratumoral administration of recombinant human interleukin 12 in head and neck squamous cell carcinoma patients elicits a T-helper 1 profile in the locoregional lymph nodes. Clin Cancer Res.

[25] Fallon J (2014). The immunocytokine NHS-IL12 as a potential cancer therapeutic. Oncotarget.

[26] Pasche N (2012). The antibody-based delivery of interleukin-12 to the tumor neovasculature eradicates murine models of cancer in combination with paclitaxel. Clin Cancer Res.

[27] Mansurov A (2020). Collagen-binding IL-12 enhances tumour inflammation and drives the complete remission of established immunologically cold mouse tumours. Nat Biomed Eng.

[28] Lewis ND (2021). Exosome surface display of IL-12 results in tumor-retained pharmacology with superior potency and limited systemic exposure compared to recombinant IL-12. Mol Cancer Ther.

[29] Zaharoff DA (2010). Intratumoral immunotherapy of established solid tumors with chitosan/IL-12. J Immunother.

[30] Daud AI (2008). Phase I trial of interleukin-12 plasmid electroporation in patients with metastatic melanoma. J Clin Oncol.

[31] Hewitt SL (2020). Intratumoral interleukin-12 mRNA therapy promotes TH1 transformation of the tumor microenvironment. Clin Cancer Res.

[32] Li Y (2020). Multifunctional oncolytic nanoparticles deliver self-replicating IL-12 RNA to eliminate established tumors and prime systemic immunity. Nat Cancer.

[33] Agarwal Y (2022). Intratumourally injected alum-tethered cytokines elicit potent and safer local and systemic anticancer immunity. Nat Biomed Eng.

[34] Wittrup KD (2022). Intratumorally anchored cytokine therapy. Expert Opin Drug Deliv.

[35] HogenEsch H (2018). Optimizing the utilization of aluminum adjuvants in vaccines: you might just get what you want. NPJ Vaccines.

[36] Flarend RE (1997). In vivo absorption of aluminium-containing vaccine adjuvants using 26Al. Vaccine.

[37] Tagliabracci VS (2015). A single kinase generates the majority of the secreted phosphoproteome. Cell.

[38] Morefield GL (2005). Effect of phosphorylation of ovalbumin on adsorption by aluminum-containing adjuvants and elution upon exposure to interstitial fluid. Vaccine.

[39] Moyer TJ (2020). Engineered immunogen binding to alum adjuvant enhances humoral immunity. Nat Med.

[40] Chen J (2014). Comparison of succinimidyl [(125)I]iodobenzoate with iodogen iodination methods to study pharmacokinetics and ADME of biotherapeutics. Pharm Res.

[41] Yazaki PJ, Bet al (2013). A series of anti-CEA/anti-DOTA bispecific antibody formats evaluated for pre-targeting: comparison of tumor uptake and blood clearance. Protein Eng Des Sel.

[42] Hasan M (1999). IL-12 is a heparin-binding cytokine. J Immunol.

[43] Stephen J (2017). Neutrophil swarming and extracellular trap formation play a significant role in Alum adjuvant activity. NPJ Vaccines.

[44] Zhang L (2020). Single-cell analyses inform mechanisms of myeloid-targeted therapies in colon cancer. Cell.

[45] Hong Y (2022). Cure of syngeneic carcinomas with targeted IL-12 through obligate reprogramming of lymphoid and myeloid immunity. JCI Insight.

[46] Garcia-Diaz A (2017). Interferon receptor signaling pathways regulating PD-L1 and PD-L2 expression. Cell Rep.

[47] Momin N (2022). Maximizing response to intratumoral immunotherapy in mice by tuning local retention. Nat Commun.

[48] Park CG (2018). Extended release of perioperative immunotherapy prevents tumor recurrence and eliminates metastases. Sci Transl Med.

