# Supplementary material for: Intratumoral aluminum hydroxide–anchored IL-12 drives potent antitumor activity by remodeling the tumor microenvironment
Source: JCI Insight. 2023 Dec 8;8(23):e168224. doi: 10.1172/jci.insight.168224 (PMC10795832; doi:10.1172/jci.insight.168224)
Supplement: undefined [file jciinsight-8-168224-s075.pdf]

Supplementary Materials for

Intratumoral aluminum hydroxide-anchored IL-12 drives potent anti-tumor activity by remodeling the tumor microenvironment

Sailaja Battula[1*], Gregory Papastoitsis[1], Howard L. Kaufman[1], K. Dane Wittrup[2], Michael M. Schmidt[1]

[*]Corresponding author. Email: sbattula@ankyratx.com

Contents:

Materials and Methods

Fig. S1. Alhydrogel® retention and activity of optimized mouse IL-12-ABP proteins

Fig. S2. Tumor retention and tissue biodistribution of ^{125}I labeled mANK-101

Fig. S3. IV clearance and heparin binding of IL-12-ABP and IL-12 proteins

Fig. S4. Alum does not have therapeutic or immunologic activity

Fig. S5. Nanostring analysis of mANK-101 induced tumor changes

Fig. S6. Changes in overall cell populations after mANK-101 treatment by scRNA-seq

Fig. S7. mANK-101 combination activity with CTLA-4 blockade

Fig. S8. T cell subclustering by scRNA-seq

Table S1. Retention time and conductivity of IL-12 or IL-12-ABP proteins from Heparin HP HiTrap column during gradient from 0 to 1.5 M NaCl in 50 mM HEPES, pH 7.4.

Table S2. Serum cytokines from cynomolgus macaques treated with ANK-101

Table S3. SPECT/CT acquisition and reconstruction parameters

Table S4. FACS antibody reagents

Table S5. Genes used in Nanostring cell signatures

Table S6. Hematology parameters measured in cynomolgus macaque study

Table S7. Clinical chemistry parameters measured in cynomolgus macaque study

Supplementary Materials and Methods

Cloning

Codon optimized DNA sequences were synthesized at ATUM Bio (Newark, CA). Human Fam20C (Uniprot Q8IXL6) was cloned into the PD2610-v10 transient transfection vector with its native signal peptide and a linker-KDEL sequence (GGGSKDEL) for intracellular retention fused at the c-terminus. Single-chain human IL-12 constructs were cloned with a human IL2 signal peptide and mature human IL-12B/p40 (Uniprot P29460) fused to mature human IL-12A/p35 (Uniprot P29459) through a $(G_4S)_3$ linker. Single-chain mouse IL-12 constructs were cloned with a human IL2 signal peptide and mature mouse IL-12B/p40 (Uniprot P43432) fused to mouse IL-12A/p35 (Uniprot P43431) through a $(G_4S)_3$ linker. For constructs containing an alum binding peptide (ABP), IL-12 sequences were fused at the C-terminus to DNA encoding a linker GGGGEGGGG followed by 8 repeats of the sequence SEEGGGG. Constructs for human IL-12, mouse IL-12, and mouse IL-12-ABP were further appended with a c-terminal His-6 tag and cloned into a pD2610-v10 vector for transient transfection. The human IL-12-ABP gene was cloned without the c-terminal His-tag, instead ending in a single serine residue. Human IL-12-ABP and Fam20C-KDEL constructs were cloned into a single ATUM Leap-In-Transposase vector with two expression cassettes with different promoters selected to express the hIL-12-ABP polypeptide at an 8-fold higher level than Fam20C-KDEL.

Protein expression and purification

Plasmids encoding human IL-12, mouse IL-12, or mouse IL-12-ABP were transiently transfected in suspension HEK-293 cells either with or without co-transfection with the human Fam20C-KDEL plasmid. In most co-transfections, a 4:1 mass ratio of IL-12 plasmid to Fam20C-KDEL plasmid was used. Supernatants were harvested and IL-12 fusion polypeptides purified by affinity chromatography on NiSepharose Excel (Cytiva 17-3712-02). Eluted fusion polypeptides were formulated in Tris-buffered saline (TBS), pH 7.4 and purity determined on a Perkin Elmer GXII capillary electrophoresis system. Polypeptide aggregation was assessed by HPLC-SEC with a 300Å pore size. Where needed, proteins were further purified by FPLC-SEC on a HiLoad 16/600 or 26/600 Superdex 200 pg column (Cytiva 28-9893-36) and monomeric peak fractions were pooled. IL-12-ABP polypeptides were further polished by anion exchange chromatography to enrich for highly phosphorylated species. Fusion polypeptides in TBS were

diluted 3-fold in WFI and loaded on a HiTrap Q Sepharose column. Samples were washed with 1% Tx-13 in 0.33x TBS followed by 15 column volumes of 20 mM Tris, pH 8.1 then eluted with a linear gradient from 20 mM Tris, pH 8.1 to 20 mM Tris, pH 8.1 + 600 mM NaCl over 20 column volumes. Selected fractions were pooled and buffer exchanged into TBS. Human IL-12-ABP proteins were stably transfected in CHO cells using the ATUM Leap-In-Transposase system and stable pools selected. After 14-day expression in a fed-batch culture, supernatants were harvested and purified by multiple chromatography steps.

HEK-Blue-IL-12 reporter assay

In vitro IL-12 signaling activity was assessed using the HEK-Blue-IL-12 reporter assay (Invivogen hkb-il12) according to manufacturer's instructions. This cell line is derived from HEK293 cells stably transfected with human IL-12Rβ1 and hIL-12Rβ2 and a secreted alkaline phosphatase (SEAP) reporter under the control of a STAT4 inducible promoter. Since mouse IL-12 cross-reacts with the human IL-12 receptors, this cell line can be used to assess potency of both human and mouse IL-12 derived constructs. HEK-Blue-IL-12 cells were cultured in DMEM + 4.5 g/L glucose, 2 mM L-glutamine, 10% heat inactivated FBS, Pen-Strep (100 U/mL) and 100 μg/mL Normocin and passaged at 70-80% confluency. For potency testing, the same media was used without Normocin.

Human or mouse IL-12-ABP proteins in TBS were mixed with Alhydrogel® to a final concentration of 200 μg/mL fusion polypeptide and 2 mg/mL aluminum hydroxide, then incubated at RT for 30 minutes. Mixtures were diluted in elution buffer containing a final concentration 1 mM phosphate and 20% serum and incubated at 37°C with rotating for 24 hours. Samples were centrifuged at 18,000xg at 4°C for 10 minutes to pellet alum and the supernatant carefully removed and saved. Pellets were resuspended in an equal volume of elution buffer. Samples were diluted in assay media to generate a titration series with a top concentration of 10 μg/mL and 3x dilutions. 20 μL of each sample in the titration series was transferred to a 96 well plate and mixed with 180 μL of HEK-Blue-IL-12 cell suspension (280,000 cells/mL) for a final top fusion polypeptide concentration of 1 μg/mL and 50,000 cells/well. Plates were then incubated overnight at 37°C in 5% CO_2. The next day, 20 μL of supernatant from each well was transferred to a new plate and mixed with 180 μL of QUANTI-Blue solution (Invivogen rep-

qbs), a colorimetric reagent that turns blue in the presence of secreted alkaline phosphatase. Plates were incubated for at 37°C for 3 hours, then absorbance measured at 620-655 nm.

^{125}I protein labeling

2 µL of Na ^{125}I (Perkin Elmer) in 0.1 M NaOH was added to a sterile 1.5 mL Axygen vial. In order, 50 µL of 3% acetic acid in methanol, 10 µL of 2.5 mg/mL N-Succinimidyl-3-(tri-n-butylstannyl) benzoate (ATE) in methanol, and 10 µL of 2.0 mg/mL N-Chlorosuccinimide (NCS) in methanol were added to the vial to form N-succinimidyl-3-[^{125}I]iodobenzoate (^{125}I-SIB). The reaction was allowed to proceed at room temperature for 5 minutes then quenched with 15 µL of 2.0 mg/mL sodium bisulfate. Reaction mixtures were loaded on a Waters 500 mg silica sep-pak cartridge pre-conditioned with 3 mL of n-hexane. Vials were rinsed with 50 µL of 80/20 n-hexane/ethyl acetate and rinse added to the packing bed. The sep-pak column was eluted with 6 mL of 80/20 n-hexane/ethyl acetate and 500 µL fractions collected. Fractions with the highest activity were pooled and concentrated to dryness in a 1.5 mL Axygen vial at 60°C under a stream of argon.

mIL-12-ABP was buffer exchanged into 0.2 M phosphate buffer, pH 7.5 using a 20 K Slide-A-Lyzer dialysis device. 119 µg of mIL-12-ABP in 50 µL was mixed with the ^{125}I-SIB reagent resuspended in 150 µL of 200 mM phosphate buffer, pH 7.5. The reaction was incubated in an Eppendorf Thermomixer C at 37°C and 300 RPM. After 60 minutes, samples were transferred to a 10K Amicon centrifuge filter and washed five times with 40 mM Tris-HCl, 150 mM sodium chloride, pH 7.5 with concentration at 10,000 rcf for 5 minutes. The final specific activity of the ^{125}I-conjugated mIL-12-ABP was 9.0 µCi/µg with a radio concentration of 7.1 µCi/µL. Cold mIL-12-ABP test article was added to the sample to adjust the specific activity concentration to 5.0 µCi/µg.

SPECT/CT image analysis

SPECT and CT images were acquired using acquisition and reconstruction parameters described in **Table S3**. Reconstructed images were generated in units of activity with values assigned to the voxels comprising the 3D reconstructed SPECT images in units of µCi.

Reconstructed images were co-registered to one another, resampled to 0.2 mm^3 voxels, and cropped to a uniform size prior to analysis. Whole body region of interest (ROI) was generated by CT-based thresholding with manual alterations. Thyroid ROI was defined by placing a single fixed volume sphere to the respective organ in each image. Liver ROI was defined by placing two fixed volume spheres in the anatomical area as defined by the CT. Kidney ROI was defined by placing phantom organs over the kidneys. Bladder ROI was defined by thresholding to encompass all activity in the area. In cases where there was no activity, one fixed volume sphere was placed in the corresponding anatomical location. The tumor ROI was manually generated based on the tumor boundaries visualized on the CT.

Results were presented in units of percent injected dose (%ID) or percent injected dose per gram (%ID/g). The %ID for each analyzed region from the *in vivo* imaging data or *ex vivo* gamma count data can be defined as %ID = Uptake/Injected Dose * 100 where Uptake = radioactivity (μCi) in a particular ROI or gamma counting sample, decay corrected to the time of injection and Injected Dose = radioactivity (μCi) injected into the subject. The %ID/g for each analyzed region from the *in vivo* imaging data or *ex vivo* gamma count data can be defined as %ID/ROI_weight where ROI_weight for imaging based data is calculated as the volume of the particular ROI in mL assuming a tissue density of 1 g/mL and for *ex vivo* gamma count data is the sample weight of the tissue.

Mouse splenocyte potency assay

Spleens were isolated from C57BL/6 mice and kept in RPMI 1640 media. To isolate splenocytes, spleens were gently mashed through a 70 μm strainer using the plunger of a 5 mL sterile syringe then washed with 5 mL RPMI 1640 media. Cells were pelleted and resuspended in 1 mL red blood cell (ACK) lysing buffer for 2 min at RT, washed multiple times, and resuspended in complete media (RPMI 1640 with 10% FBS, 1% P/S and 55 μM β-ME). Splenocytes were plated in 96 well round bottom plates at a density of $5x10^5$ cells per well and stimulated 100 ng/mL anti-CD3 (clone 145-2C11) in the presence of vehicle or a titration of free mIL-12-ABP or mANK-101 complex. Following a 72-hour incubation, cell culture supernatants were harvested and analyzed for IFNγ using a Mouse IFN-γ Immunoassay Quantikine ELISA Kit (R&D Systems).

Heparin binding assay

Human or mouse IL-12 or IL-12-ABP proteins in 50 mM HEPES, pH 7.4 were loaded onto a 1 mL Heparin HP HiTrap column at a 0.5 mL/min flow rate. Bound protein was eluted with a gradient from equilibration buffer A (50 mM HEPES, pH 7.4) to elution buffer B (50 mM HEPES, pH 7.4 + 1.5 M NaCl) over 20 minutes.

Cynomolgus macaque toxicology study

Naïve cynomolgus macaques, age 2.5 – 5 years, were administered a single subcutaneous administration of 0.2, 2, or 20 µg/kg ANK-101 on day 1 or two repeat administrations of 2 or 20 µg/kg ANK-101 on days 1 and 8 with two animals (1/sex) per group. Clinical observations, injection site observations, body weights, and body temperatures were recorded. For coagulation measurements, blood samples were collected with sodium citrate anticoagulant pre-dose and days 2 and 8 for the single-dose groups or pre-dose and day 15 for the repeat dose groups. Prothrombin time (PT) and activated partial thromboplastin time (APTT) were measured by solidification method using a Sysmex coagulation analyzer. For hematology, blood was collected with EDTA-K_2 anticoagulant pre-dose and on days 2 and 8 for the single-dose groups and pre-dose and days 2, 7, 9, and 15 for the repeat dose groups. Samples were run on an ADVIA 2120 hematology system with detected parameters listed in Supplemental **Table 5**. For clinical chemistry, serum was prepared from blood samples taken pre-dose and on days 2 and 8 for the single-dose groups and pre-dose and days 2, 7, 9, and 15 for the repeat dose groups and analyzed on a TBA-120FR clinical chemistry analyzer for parameters listed in Supplemental **Table 6**. For systemic cytokine measurements, serum was prepared from blood samples drawn at pre-dose, 2 hours, 1 day, or 3 days after each dose, and on day 8 or 15 for the single or repeat dose groups respectively and analyzed by MSD using kit K15068L to quantify IL-2, IL-6, IL-10, TNF-α, IL-1β, IL-12p70, IFN-γ, and CXCL10.

Study Approval

All procedures involving the care and use of animals in this study were performed according to the guidelines approved by Institutional Animal Care and Use Committee (IACUC) at JOINN LABORATORIES (Suzhou) Inc. Animal care was compliant with the SOPs of JOINN

LABORATORIES (Suzhou) Inc., the Guide for the Care and Use of Laboratory Animals (8th Edition, Institute of Laboratory Animal Resources, Commission on Life Sciences, National Research Council; National Academy Press; Washington, D.C., 2011), and the U.S. Department of Agriculture through the Animal Welfare Act (Public Law 99-198). JOINN LABORATORIES (Suzhou) Inc. is fully accredited by the Association for Assessment and Accreditation of Laboratory Animal Care International (AAALAC).

Supplemental Figure 1

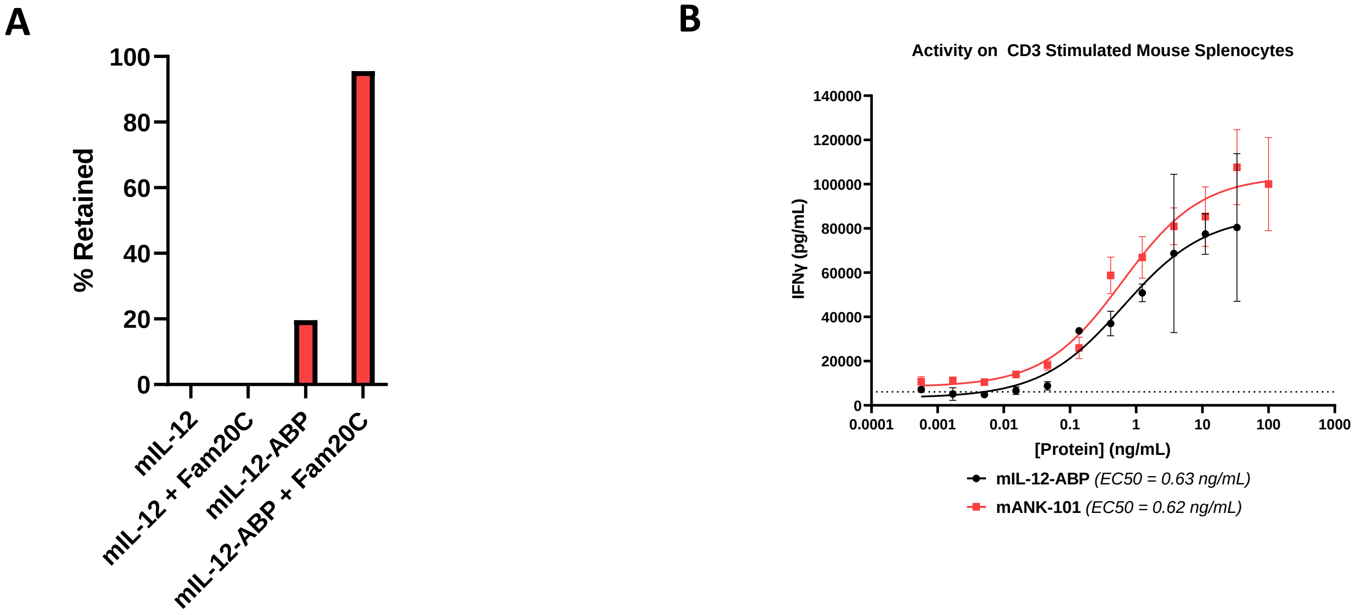

Supplemental Figure 1. Alhydrogel® retention and activity of optimized mouse IL-12-ABP proteins. (**A**) Percent of Alhydrogel® complexed IL-12-ABP remaining bound during incubation in Tris-buffered saline (TBS) with 20% serum as measured by free protein detected in supernatant by ELISA. (**B**) IFNγ production measured by ELISA from activated murine splenocytes following incubation with a titration of free mIL-12-ABP protein or mANK-101 complex.

Supplemental Figure 2

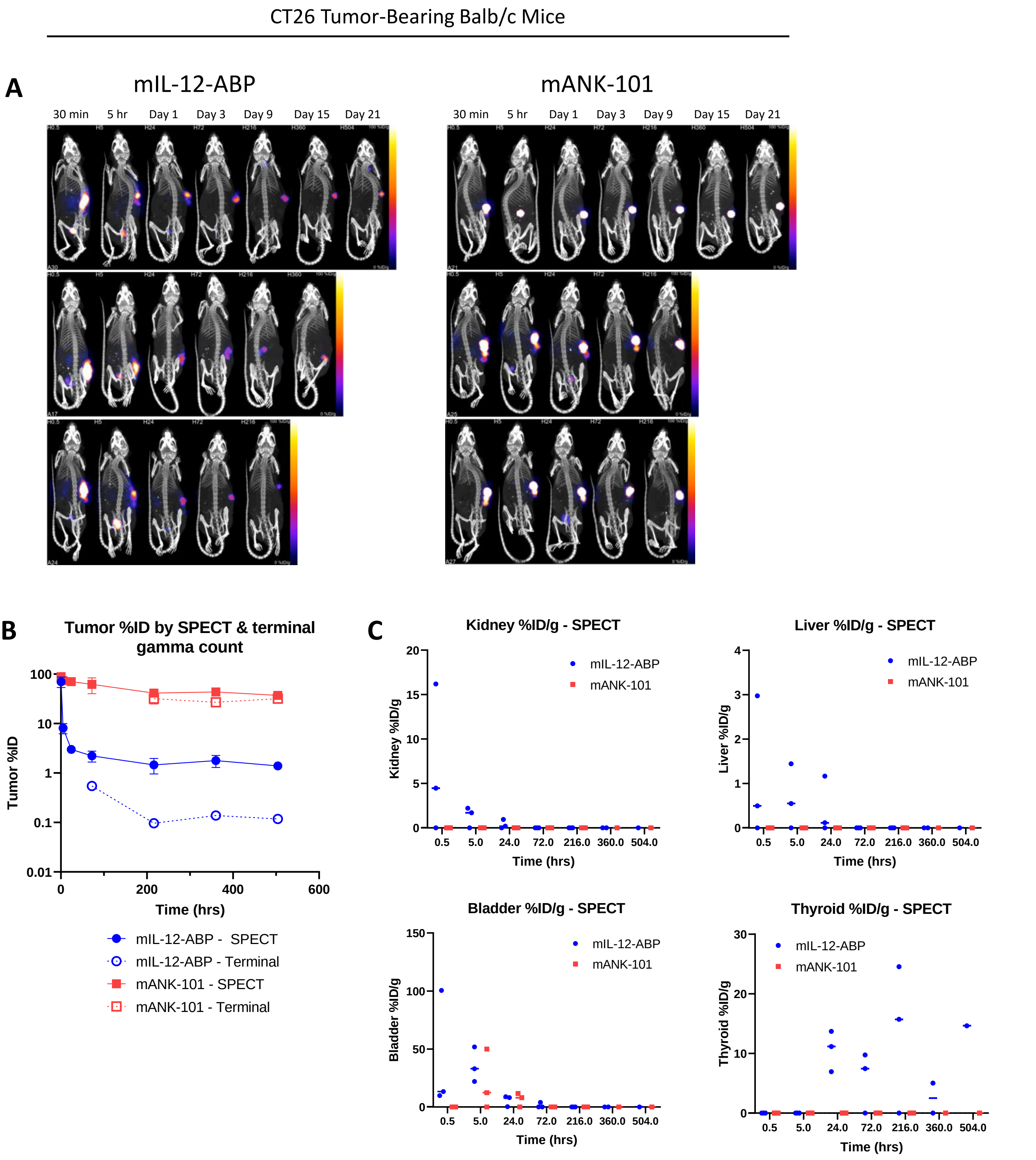

Supplemental Figure 2. Tumor retention and tissue biodistribution of ^{125}I-labeled mANK-101. (**A**) SPECT/CT images of BALB/c mice bearing CT26 tumors after a single IT administration of ^{125}I-labeled mIL-12-ABP as free protein or mANK-101 complex (also presented in Figure 2C). (**B**) Percent injected dose (%ID) in tumor of ^{125}I-labeled mIL-12-ABP or mANK-101 as measured from SPECT/CT image analysis or ex vivo gamma counts. (**C**) Percent injected dose per gram (%ID/g) in different tissues of ^{125}I-labeled mIL-12-ABP or mANK-101 as measured from SPECT/CT image analysis.

Supplemental Figure 3

A

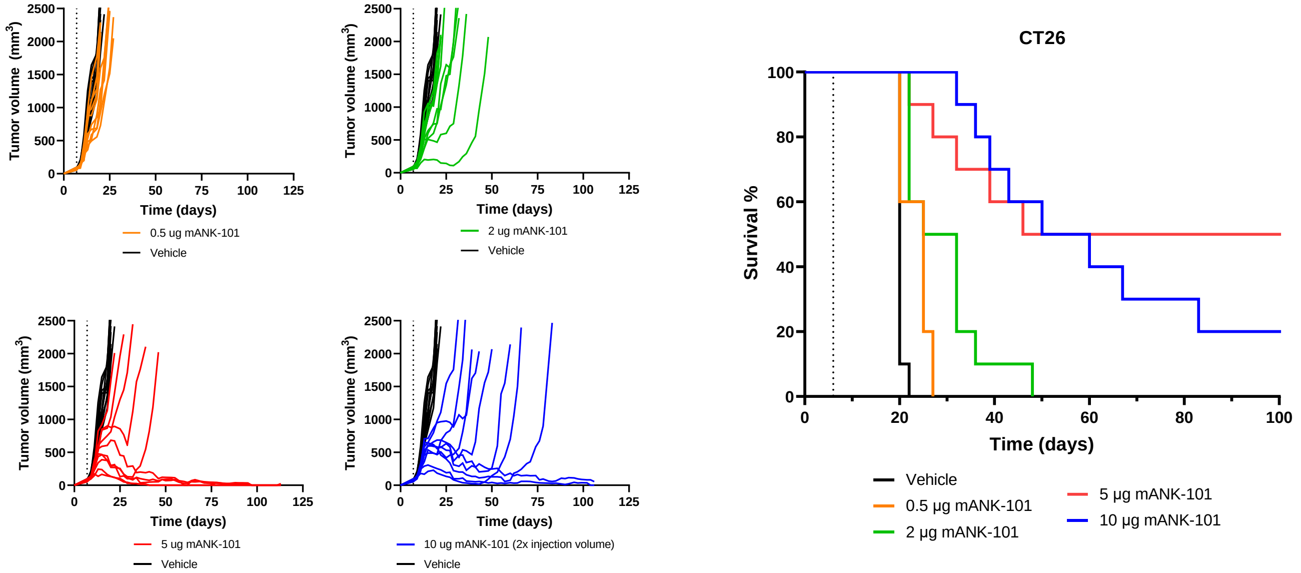

B

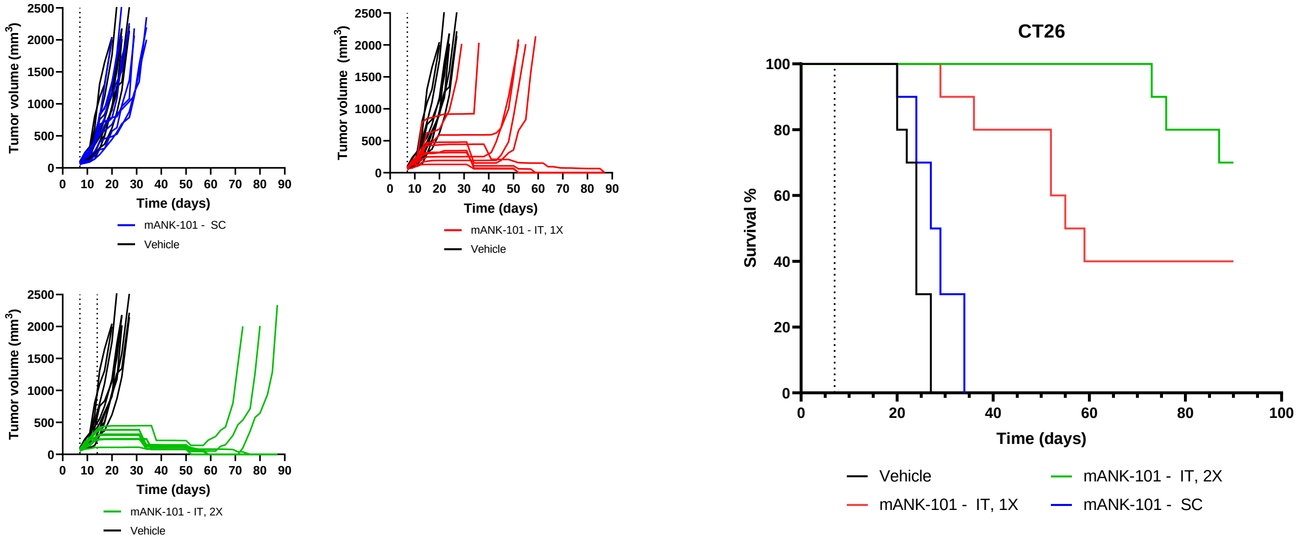

C

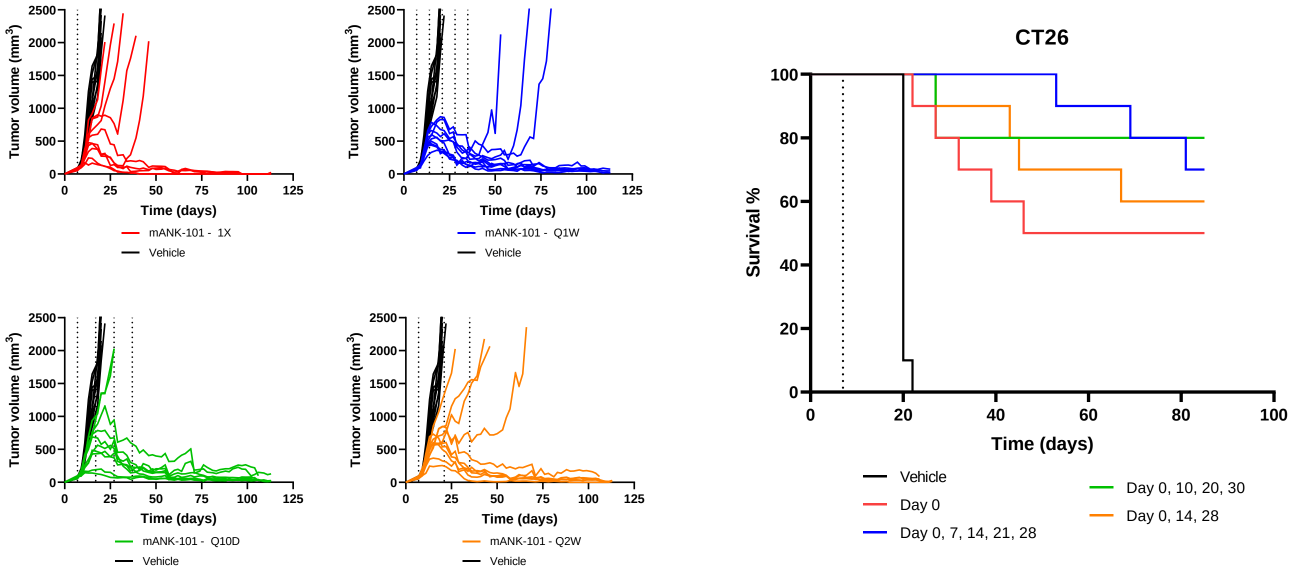

Supplemental Figure 3

D

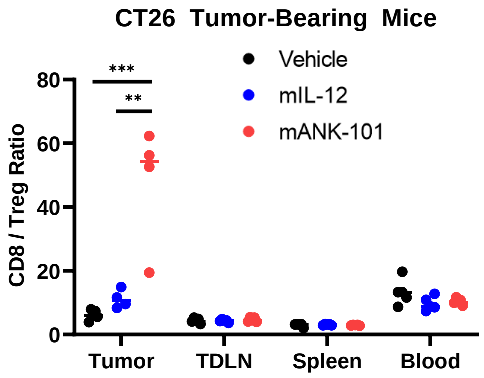

Supplemental Figure 3. Dosing optimization of mANK-101 in CT26 tumor model. (**A**) Survival curves and individual tumor volume traces from BALB/c mice (n = 10) bearing CT26 tumors treated with a single IT injection of vehicle, 0.5, 2, 5, or 10 µg mANK-101 complex. All injections were administered in a 20 µL volume, except the 10 µg group which was administered in 40 µL. The dashed vertical line denotes the treatment day. (**B**) Survival curves and individual tumor volume traces from BALB/c mice (n = 10) bearing CT26 tumors treated with 5 µg mANK-101 complex either as a single IT injection, two IT injections 1 week apart, or a single subcutaneous injection on the opposite flank. (**C**) Survival curves and individual tumor volume traces from BALB/c mice (n = 10) bearing CT26 tumors treated with 5 µg mANK-101 complex either as a single IT injection, 3 IT injections spaced 14 days apart, 4 IT injections spaced 10 days apart, or 5 IT injections spaced 1 week apart. (**D**) Mice bearing CT26 tumors (n = 5) were treated with a single IT injection of vehicle, 5 µg mIL-12, or 5 µg mANK-101 and tumors, tumor-draining lymph node (TDLN), spleen, and peripheral blood analyzed on Day 7 by FACS. Ratio of CD8 + /Foxp3+ T cells. One mouse in mIL-12 and ANK-101 groups was removed from tumor analysis due to lymph node contamination. FACS analysis of tumors, TDLN, spleen, and blood was performed on Day 7. FACS comparisons performed by one-way ANOVA with Tukey's post-test *P<0.05, **P<0.005, ***P<0.0005.

Supplemental Figure 4

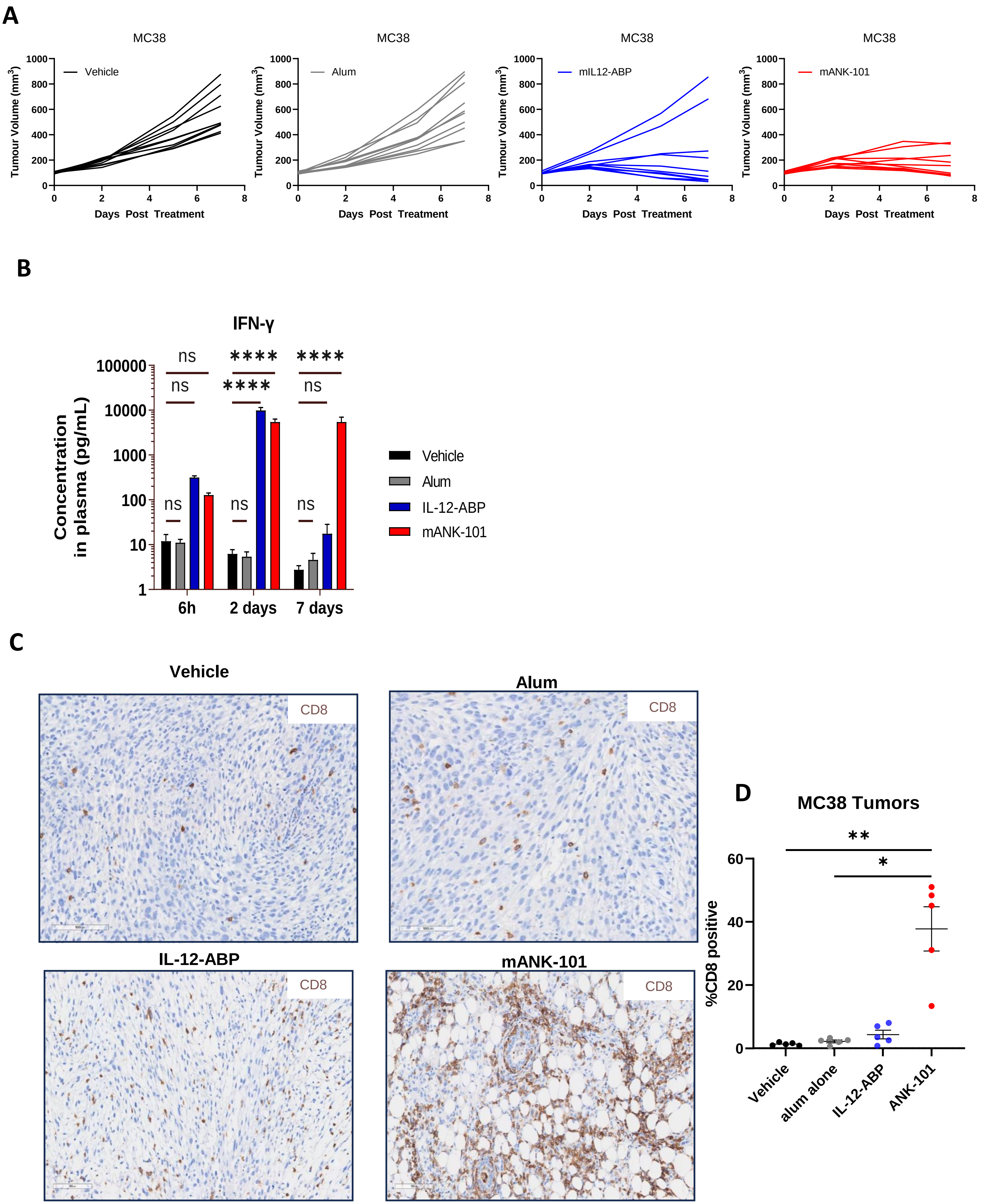

Supplemental Figure 4. Alum does not have therapeutic or immunologic activity. (**A**) Tumor growth from mice (n = 10) bearing MC38 tumors (100 mm^3) were treated with a single IT injection of vehicle, IL-12-ABP alone (5µg), alum (50µg) or a combination of IL-12-ABP + alum. (**B**) In the same experiments, serial plasma collections were carried out for systemic IFNγ levels. (**C**) CD8+ T cell immunohistochemistry of MC38 tumors (n=5) following a single IT injection of vehicle or 50 µg of alum alone 7 days post-treatment. Representative images (left) with the quantification of %CD8+ T cells positives in vehicle and alum treated groups (right). IHC comparisons performed by one-way ANOVA with Tukey's post-test *P<0.05, **P<0.005.

Supplemental Figure 5

A

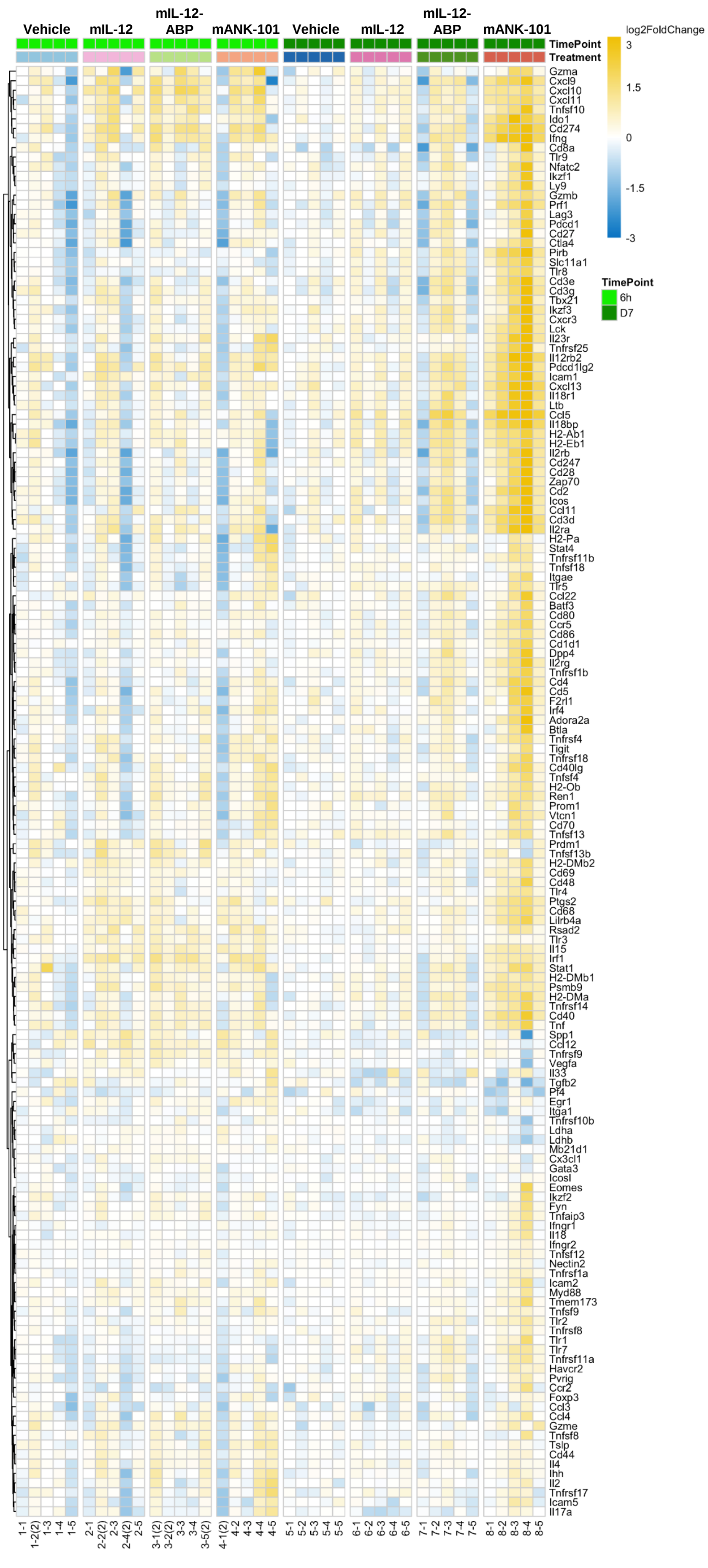

B

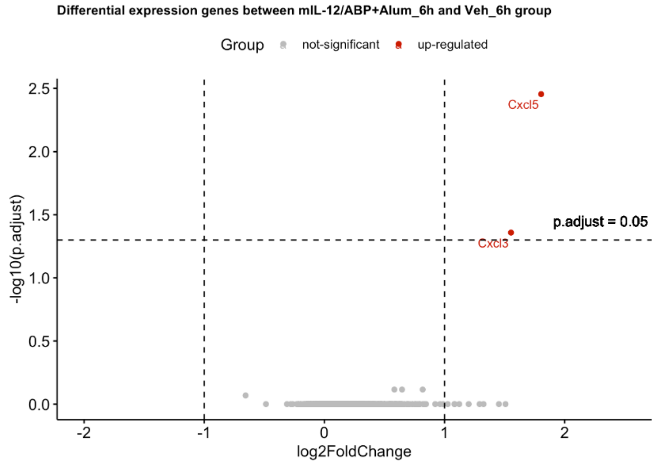

C

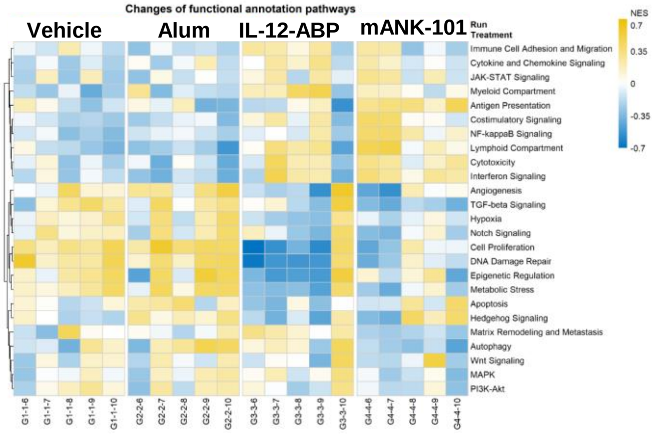

Supplemental Figure 5. Nanostring analysis of mANK-101 induced tumor changes. (**A**) Heatmap representing changes in relative expression of genes related to T cell Priming and Activation in MC38 tumors either 6 hours or 7 days after a single IT administration of vehicle, 4.6 µg mIL-12, 5 µg free mIL-12-ABP protein, or 5 µg mANK-101 as measured with Nanostring Mouse PanCancer IO360 Panel. (**B**) Differentially expressed genes between mANK-101 and vehicle treated mice at 6 hours post-treatment. (**C**) In a separate set of experiments, alum alone control was included and intratumoral gene expression was analyzed on day 7 after treatment by Nanostring Mouse PanCancer IO360 Panel (n=5).

Supplemental Figure 6

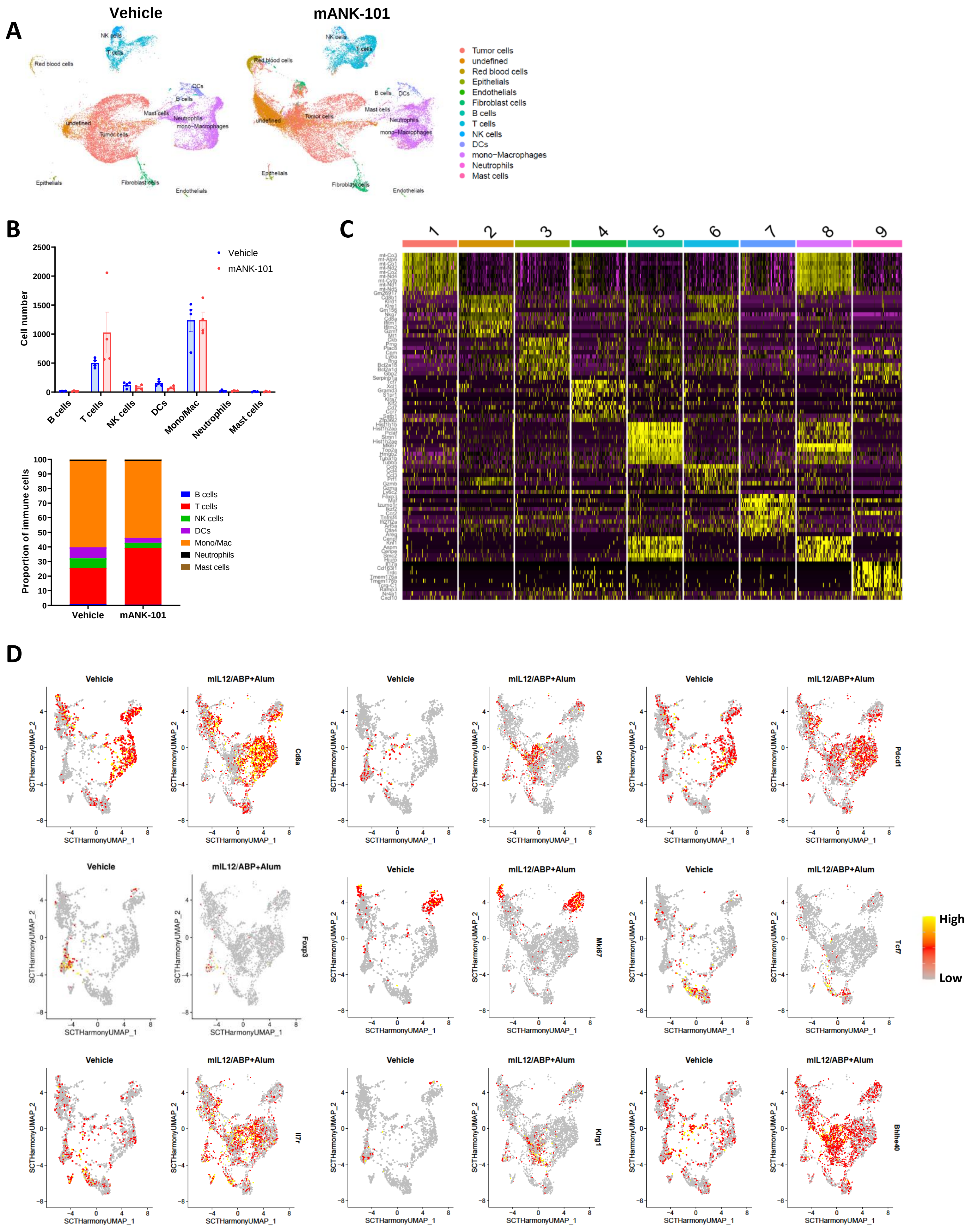

Supplemental Figure 6. Changes in overall cell populations and T cell subclustering after mANK-101 treatment by scRNA-seq. (**A**) UMAP plot of all cells in Vehicle or mANK-101 treated mice. Cells forming discrete clusters were manually annotated based on well-defined hematopoietic cell subset expression markers. (**B**) Bar graphs of the total numbers of cells (adjacent bars) or proportions (stacked bars) found in indicated hematopoietic cell subsets in vehicle-treated or mANK-101-treated mice. (**C**) Heatmap of transcripts most differentially expressed between each of the nine unsupervised snn clusters found in the T cell subsets. (**D**) Relative expression of indicated transcripts in regions of UMAP plots of T cells from vehicle or mANK-101 treated mice. Color represents relative expression.

Supplemental Figure 7

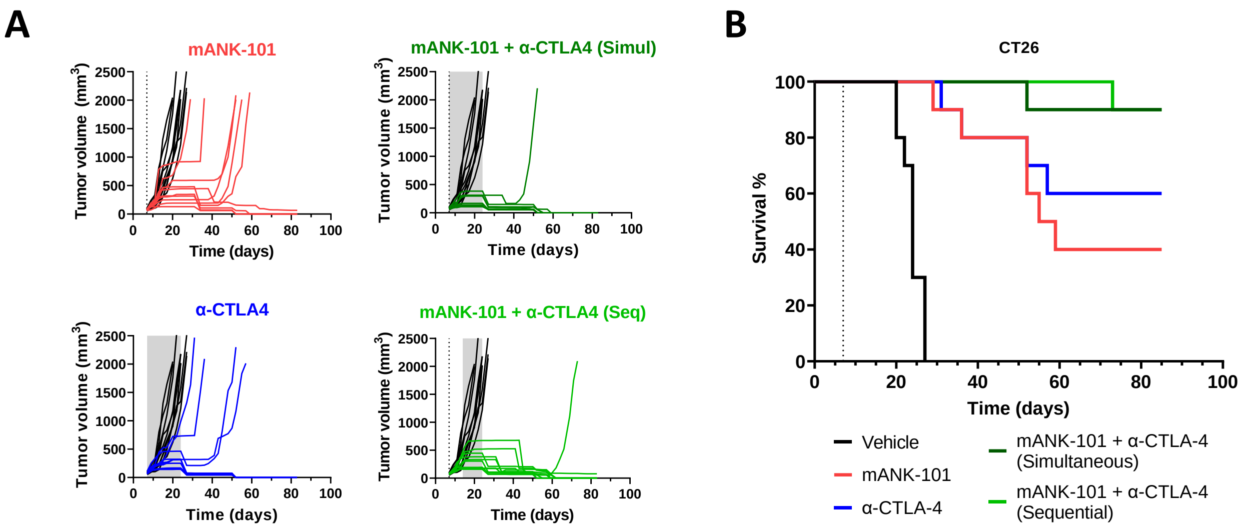

Supplemental Figure 7. mANK-101 combination activity with CTLA-4 blockade. Kaplan-Meier survival curves and individual tumor volume traces from BALB/c mice (n = 10) bearing CT26 tumors treated with a single IT injection of vehicle or 5 µg mANK-101 alone or in combination with anti-mouse CTLA-4 dosed at 200 µg IP biweekly for up to 3 weeks. Anti-CTLA-4 treatment started on the same day as mANK-101 administration (simultaneous) or 1 week later (sequential). The dashed vertical line denotes the mANK-101treatment day, and the shaded grey area represents the duration of anti-CTLA-4 treatment.

Supplemental Figure 8

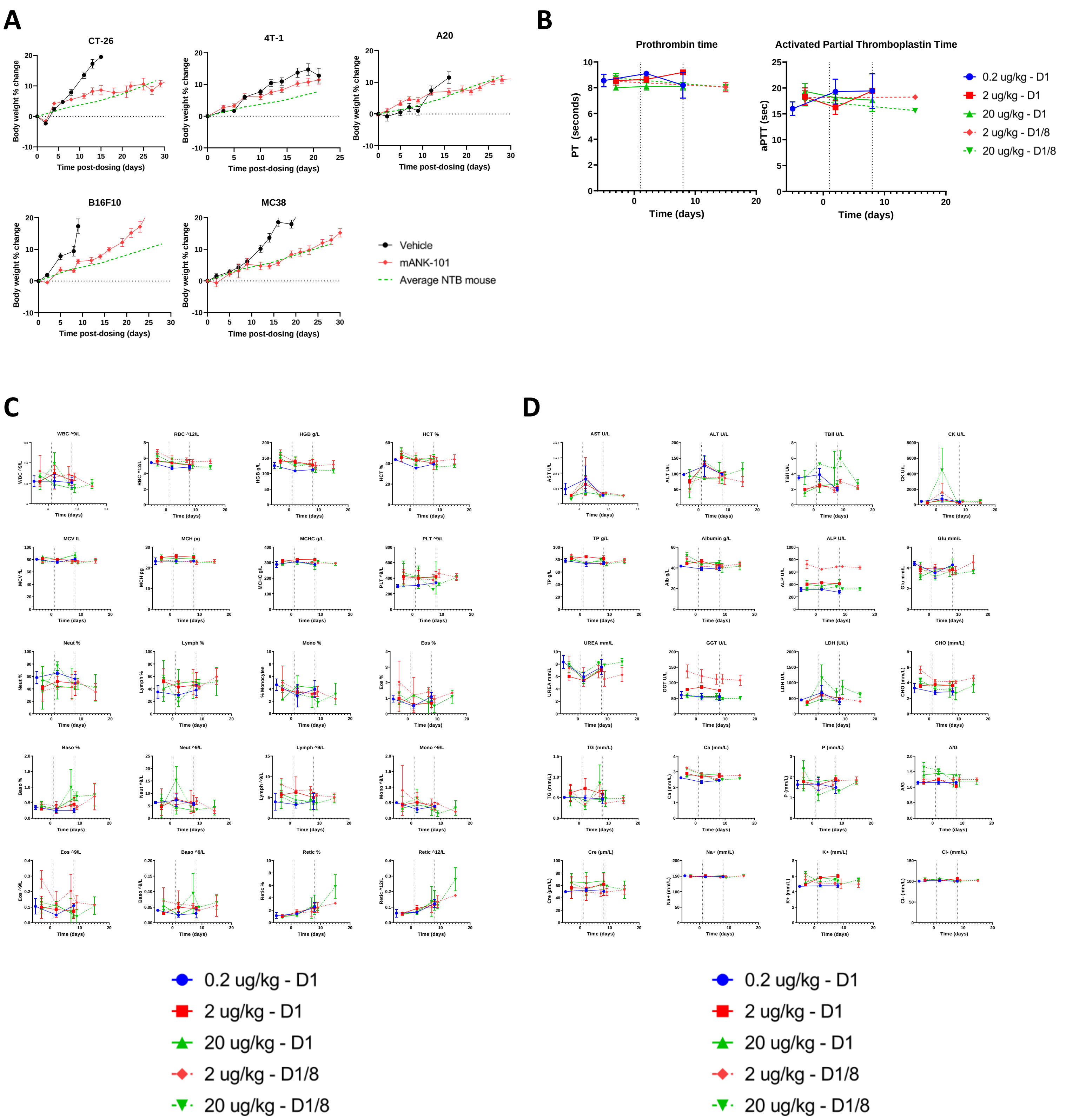

Supplemental Figure 8. Safety profiling of ANK-101 in mice and cynomolgus macaques. (**A**) Body weights of vehicle or mANK-101 treated mice in efficacy studies reported in Figure 3 compared to average growth of a non-tumor bearing (NTB) mouse. (**B**–**D**) Safety measurements from cynomolgus macaques treated with subcutaneous administration of 0.2, 2, or 20 µg/kg ANK-101 as single dose or two repeat doses 1 week apart. Vertical dashed lines represent the timing of mANK-101 administration. (**B**) Prothrombin time and activated partial thromboplastin time measured by solidification method (**C**) Hematology values measured from whole blood (**D**) Clinical chemistry values measured in serum.

Table S1.

Construct	RT (mL)	Conductivity (mS/cm)
mIL-12	12.13	39.72
mIL-12-ABP	10.86	32.97
hIL-12	11.31	36.49
hIL-12-ABP	10.20	29.32

Table S2. Serum cytokines from cynomolgus macaques treated with ANK-101

Group	Animal ID	Dosing Day	Time	IFN-γ	IL-10	IL-12p70	IL-1β	IL-2	IL-6	IP-10	TNF-α
0.2 µg/kg Single dose	22-4451	D1	0h	BQL	BQL	BQL	BQL	0.646	2.20	130	BQL
			2h	BQL	BQL	BQL	BQL	0.758	1.94	151	BQL
			24h	6.61	BQL	1.88	BQL	1.01	1.48	298	BQL
			72h	8.61	BQL	2.79	BQL	1.02	1.21	324	BQL
			168h	6.41	BQL	BQL	BQL	0.956	2.45	138	BQL
	22-4452	D1	0h	BQL	BQL	BQL	BQL	0.602	3.52	64.8	BQL
			2h	BQL	BQL	BQL	BQL	BQL	3.51	59.1	BQL
			24h	BQL	BQL	BQL	BQL	BQL	3.34	107	BQL
			72h	BQL	BQL	BQL	2.15	0.838	12.9	277	BQL
			168h	BQL	BQL	BQL	BQL	0.897	5.80	252	BQL
2 µg/kg Single dose	22-4453	D1	0h	BQL	BQL	BQL	BQL	BQL	1.35	205	BQL
			2h	BQL	BQL	BQL	BQL	0.530	3.94	255	BQL
			24h	BQL	BQL	4.64	BQL	0.702	5.24	366	BQL
			72h	BQL	BQL	7.48	BQL	0.570	2.97	267	BQL
			168h	BQL	BQL	8.58	BQL	0.992	4.56	439	BQL
	22-4454	D1	0h	BQL	BQL	BQL	BQL	BQL	1.65	246	BQL
			2h	BQL	BQL	BQL	BQL	BQL	1.85	285	BQL
			24h	BQL	BQL	BQL	BQL	BQL	1.91	322	BQL
			72h	BQL	BQL	5.00	BQL	BQL	3.09	158	BQL
			168h	BQL	BQL	6.56	BQL	0.574	7.11	479	BQL
20 µg/kg Single dose	22-4455	D1	0h	BQL	BQL	BQL	BQL	0.566	0.894	356	BQL
			2h	BQL	BQL	BQL	BQL	0.626	1.06	354	BQL
			24h	BQL	BQL	4.62	BQL	0.642	4.44	571	BQL
			72h	BQL	BQL	11.4	BQL	0.586	1.18	267	BQL
			168h	BQL	BQL	45.5	BQL	0.964	3.70	1239	BQL
	22-4456	D1	0h	BQL	BQL	BQL	BQL	0.897	1.45	319	BQL
			2h	BQL	BQL	BQL	BQL	BQL	4.49	367	BQL
			24h	BQL	BQL	6.96	BQL	0.718	1.86	732	BQL
			72h	BQL	BQL	12.2	BQL	0.489	0.612	563	BQL
			168h	BQL	BQL	20.9	BQL	0.972	2.12	1211	BQL
2 µg/kg Repeat dose	22-4457	D1	0h	BQL	BQL	BQL	1.40	1.90	1.75	363	BQL
			2h	BQL	BQL	BQL	BQL	1.76	21.0	260	BQL
			24h	BQL	BQL	BQL	BQL	1.24	9.85	351	BQL
			72h	BQL	BQL	6.44	BQL	1.30	1.88	347	BQL
		D8	0h	BQL	BQL	12.0	BQL	1.10	1.41	509	BQL
			2h	BQL	BQL	11.2	BQL	1.09	2.94	514	BQL
			24h	BQL	BQL	11.1	BQL	0.913	2.23	513	BQL
			72h	BQL	BQL	23.5	BQL	1.27	2.06	498	BQL
			168h	6.79	BQL	4.24	BQL	1.44	3.69	374	BQL
	22-4459	D1	0h	BQL	BQL	BQL	BQL	1.71	1.29	423	BQL
			2h	BQL	BQL	BQL	BQL	1.41	9.26	322	BQL
			24h	BQL	BQL	4.65	BQL	0.992	21.9	690	BQL
			72h	BQL	BQL	7.68	BQL	1.26	1.65	312	BQL
		D8	0h	BQL	BQL	10.4	BQL	1.02	1.50	509	BQL
			2h	BQL	BQL	11.3	BQL	1.00	2.07	537	BQL
			24h	BQL	BQL	19.4	BQL	1.08	1.67	339	BQL
			72h	BQL	BQL	21.3	BQL	1.19	2.84	489	BQL
			168h	BQL	BQL	9.76	BQL	BQL	1.55	213	BQL
20 µg/kg Repeat dose	22-4458	D1	0h	BQL	BQL	BQL	BQL	0.809	0.886	225	BQL
			2h	BQL	BQL	BQL	1.75	0.798	12.9	142	BQL
			24h	BQL	BQL	12.9	BQL	0.916	7.85	1493	BQL
			72h	50.0	BQL	15.8	BQL	1.02	2.70	5608	BQL
		D8	0h	38.8	BQL	14.6	BQL	1.27	2.05	6519	BQL
			2h	47.3	BQL	15.2	BQL	1.21	6.77	7034	BQL
			24h	45.4	BQL	33.9	BQL	1.37	2.64	5180	BQL
			72h	398	BQL	40.1	BQL	1.33	2.42	8271	BQL
			168h	BQL	BQL	BQL	BQL	1.17	BQL	605	BQL
	22-4460	D1	0h	BQL	BQL	BQL	BQL	1.24	1.44	180	BQL
			2h	BQL	BQL	BQL	BQL	1.48	47.6	198	BQL
			24h	BQL	BQL	11.8	BQL	1.16	7.61	382	BQL
			72h	8.52	BQL	59.8	BQL	1.75	3.75	1303	BQL
		D8	0h	778	BQL	32.6	BQL	2.52	7.91	12631	BQL
			2h	625	BQL	29.9	BQL	2.32	14.0	12763	BQL
			24h	434	BQL	47.3	4.01	2.95	13.8	11244	BQL
			72h	16.0	BQL	43.7	BQL	3.52	14.7	8903	BQL
			168h	BQL	BQL	BQL	BQL	4.22	3.41	467	BQL

Table S3. SPECT/CT acquisition and reconstruction parameters

SPECT Acquisition Parameters	
System	NanoScan SPECT/CTTM (Mediso)
Scan range	Whole body
Scan duration & time points	30 m, 5 h, 24 h, 72 d, 10 d, 15 d, 21 d (30 minutes)
Energy window	28.37 20%FW keV
Number of projections	360
Time per projection	34 s
Pinhole aperture (# & diameter)	Aperture #63 (1.0 mm)
**SPECT Reconstruction Parameters**	
Smoothing	Low
Resolution	Medium
Iterations	Medium
Corrections	Attenuation and Scatter
Voxel size	1.0 × 1.0 mm

CT Acquisition Parameters	
System	NanoScan SPECT/CTTM (Mediso)
Scan range	Whole body
Tube voltage	50 kVp
Current	640 µA
Exposure time	300 ms
Number of projections	180
**CT Reconstruction Parameters**	
Algorithm	Filtered Backprojection
Filter	Cosine
Voxel size	0.250 × 0.250 mm

Table S4. FACS reagents

Marker	Fluorochrome	Clone	Cat.	Isotype	Vender
CD45	BV785	30-F11	103149	Rat IgG2b, κ	Biolegend
CD3	BUV395	17A2	740268	Rat IgG2b, κ	BD
CD4	BV421	GK1.5	100438	Rat IgG2b, κ	Biolegend
CD8	PE-eFluor610	53-6.7	61-0081-82	Rat IgG2a, κ	eBiosciences
Foxp3	PE	FJK-16s	12-5773-82	Rat IgG2a, κ	eBiosciences
CD335	BV711	29A1.4	137621	Rat IgG2a, κ	Biolegend
CD11b	BUV661	M1/70	612977	Rat IgG2b, k	BD
F4/80	BV510	BM8	123135	Rat IgG2a, κ	Biolegend
I-A/I-E	AF700	M5/114.15.2	107622	Rat IgG2b, κ	Biolegend
Ly-6G	BUV737	1A8	741813	Rat IgG2a, κ	BD
Ly-6C	FITC	HK1.4	128006	Rat IgG2c, κ	Biolegend
PD-1	BV650	J43	744546	Armenian Hamster IgG2, κ	BD
PD-L1	Percp-cy5.5	10F.9G2	124334	Rat IgG2b, κ	Biolegend
CD19	BV605	6D5	115540	Rat IgG2a, κ	Biolegend
CD86	PE-CY7	GL1	105014	Rat IgG2a, κ	Biolegend
CD103	APC	2E7	121414	Armenian Hamster IgG, κ	Biolegend
L/D	efluo780	NA	65-0865-14	NA	eBiosciences

Table S5. Genes used in Nanostring cell signatures

Cell type	Probe set
T cells	Cd3d, Cd3e, Cd3g, Cd6, Sh2d1a, Trat1
Macrophages	Cd163, Cd68, Cd84, Ms4a4a
Th1 cells	Tbx21
NK CD56dim cells	Il21r, Klr3dl1, Klr3dl2

Table S6. Hematology parameters measured in cynomolgus macaque study

Abbreviation	Parameter	Unit	Detection methods
WBC	Total leukocyte count	$\times 10^9$/L	The Basophil / Nuclear Lobularity Analysis Method+ Two-dimensional laser flow technology + Peroxidase staining method
Neut	Neutrophilic granulocyte	$\times 10^9$/L, %	Peroxidase staining method + The Basophil / Nucle ar Lobularity Analysis Method
Lymph	Lymphocyte	$\times 10^9$/L, %	Peroxidase staining method + The Basophil / Nuclear Lobularity Analysis Method
Mono	Mononuclear cell	$\times 10^9$/L, %	Peroxidase staining method + The Basophil / Nuclear Lobularity Analysis Method
Eos	Eosinophils	$\times 10^9$/L, %	Peroxidase staining method + The Basophil / Nuclear Lobularity Analysis Method
Baso	Basophilic cell	$\times 10^9$/L, %	Peroxidase staining method + The Basophil / Nuclear Lobularity Analysis Method
RBC	Erythrocyte count	$\times 10^{12}$/L	Two-dimensional laser flow technology
HGB	Hemoglobin	g/L	Total Hgb measured by cyanmethemoglobin
HCT	Hematocrit	%	Calculation based on RBC and MCV
Retic	Reticulocyte count	$\times 10^{12}$/L, %	Two-dimensional laser flow technology+ Fluorescent staining of nucleic acids
MCV	Mean corpuscular volume	fL	calculation
MCHC	Mean corpuscular hemoglobin conc.	g/L	calculation
MCH	Mean corpuscular hemoglobin	pg	calculation
PLT	Platelet	$\times 10^9$/L	Two-dimensional laser flow technology

Table S7. Clinical chemistry parameters measured in cynomolgus macaque study

Abbreviation	Parameter	Unit	Detection methods
ALT	Alanine aminotransferase	U/L	Alanine substrate method
AST	Aspartate aminotransferase	U/L	Aspartic acid substrate method
TP	Total protein	g/L	Biuret colorimrtry
Alb	Albumin	g/L	Bromocresol green method
A/G	Albumin/ globulin ratio	-	Calculated
TBil	Total bilirubin	μmol/L	Chemical oxidation
LDH	Lactate dehydrogenase	U/L	Lactic acid substrate method
ALP	Alkaline phosphatase	U/L	Kinetic method with NPP-AMP
CK	Creatine kinase	U/L	Creatine phosphocreatine substrate method
GGT	γ-glutamyl transpeptidase	U/L	GCANA substrate method
UREA	Urea	mmol/L	Urease glutamic dehydrogenase method
Cre	Creatinine	μmol/L	Sarcosine oxidase method
CHO	Total cholesterol	mmol/L	CHOD-PAP method
TG	Triglycerides	mmol/L	GPO-PAP method
Glu	Glucose	mmol/L	Hexokinase method
Ca	Calcium	mmol/L	Arsenazo III method
P	Phosphorus	mmol/L	Ultraviolet method
Na^+	Sodium ion	mmol/L	Electrode method
K^+	Potassium ion	mmol/L	Electrode method
Cl^-	Chloride ion	mmol/L	Electrode method
