# Supplementary material for: Intratumoral aluminum hydroxide–anchored IL-12 drives potent antitumor activity by remodeling the tumor microenvironment
Source: JCI Insight. 2023 Dec 8;8(23):e168224. doi: 10.1172/jci.insight.168224 (PMC10795832; doi:10.1172/jci.insight.168224)
Supplement: Supplemental data [file jciinsight-8-168224-s076.pdf]

Supplemental Figure 1

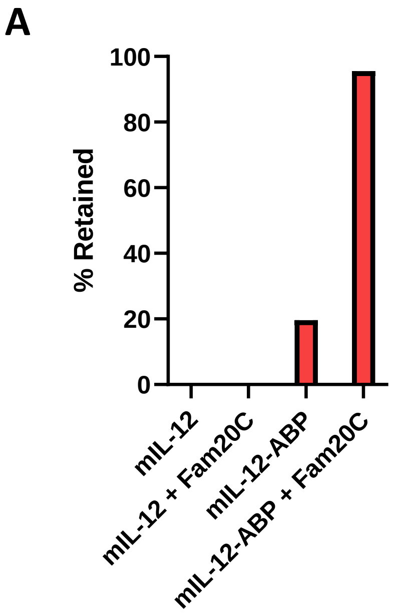

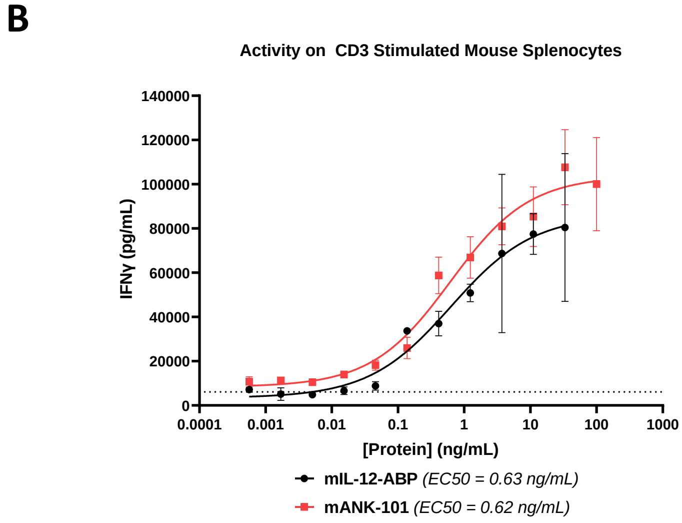

Supplemental Figure 2

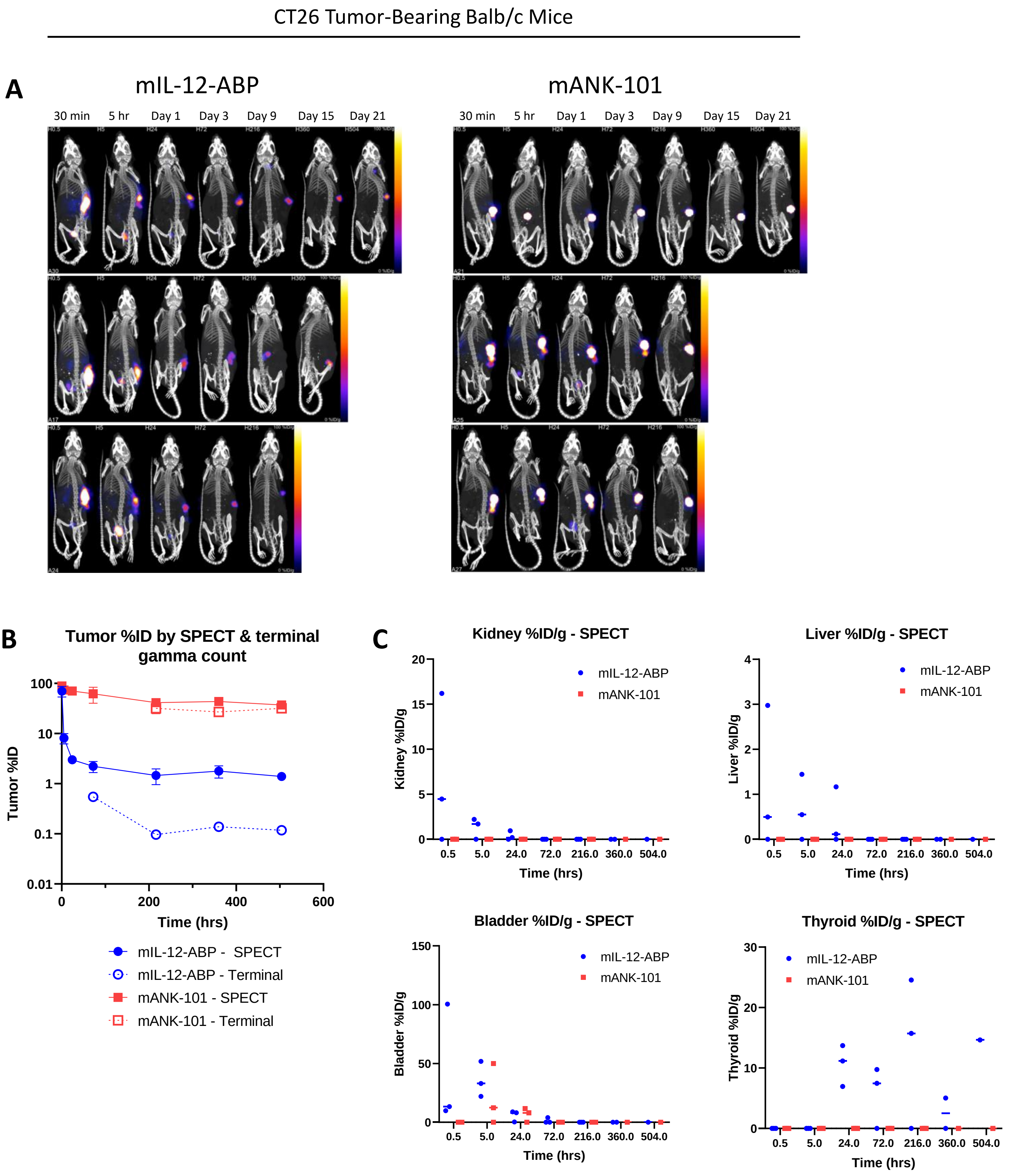

Supplemental Figure 3

A

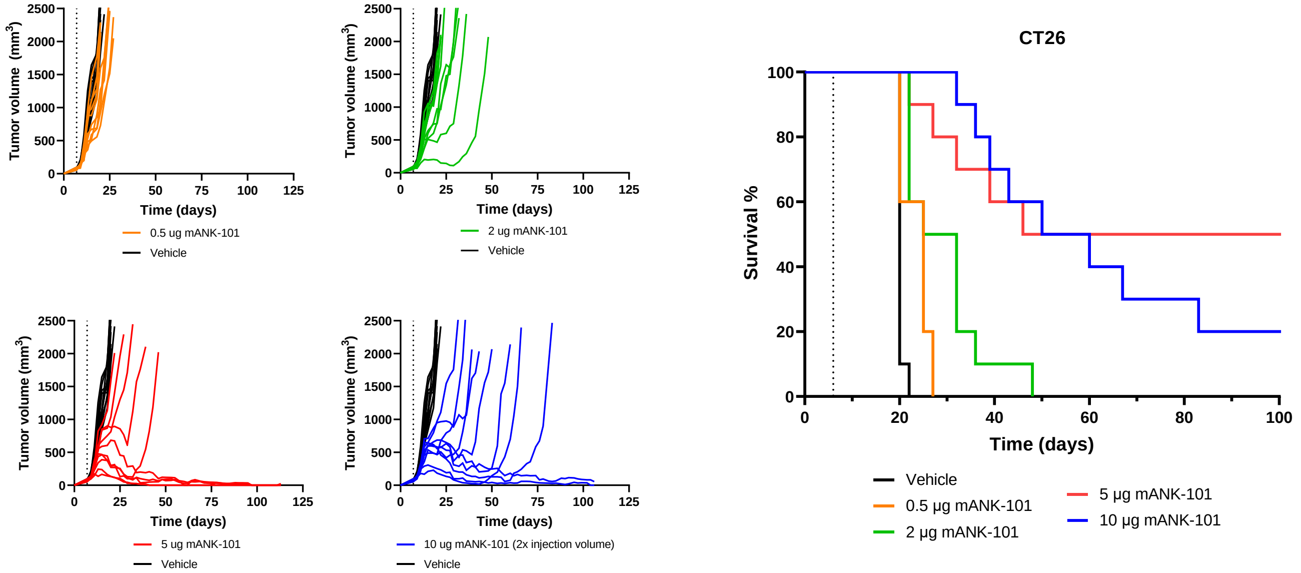

B

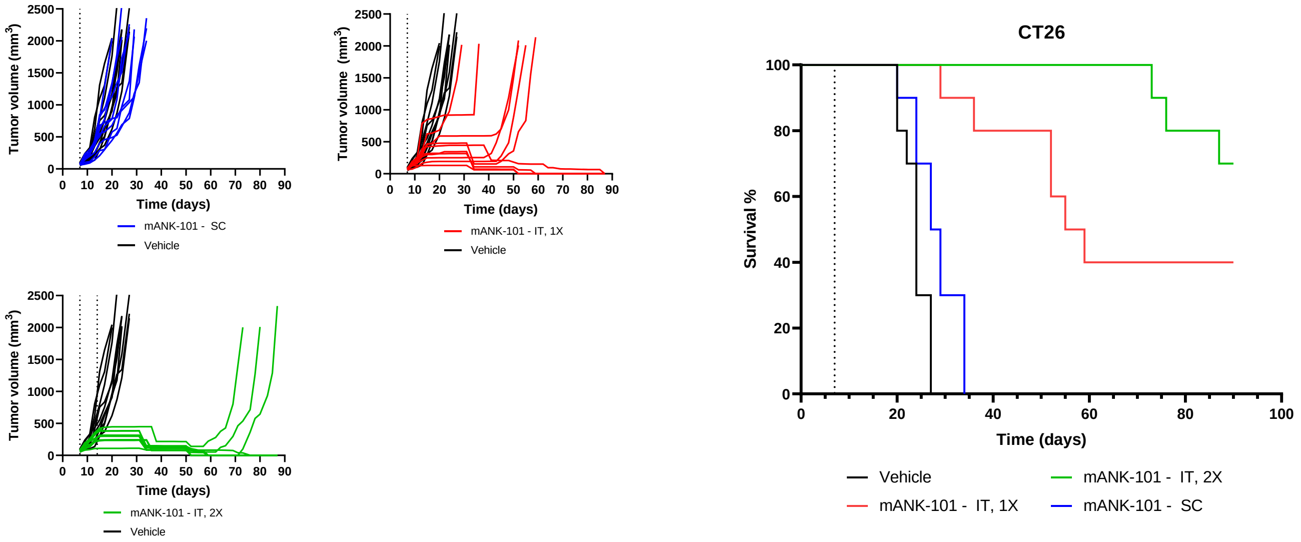

C

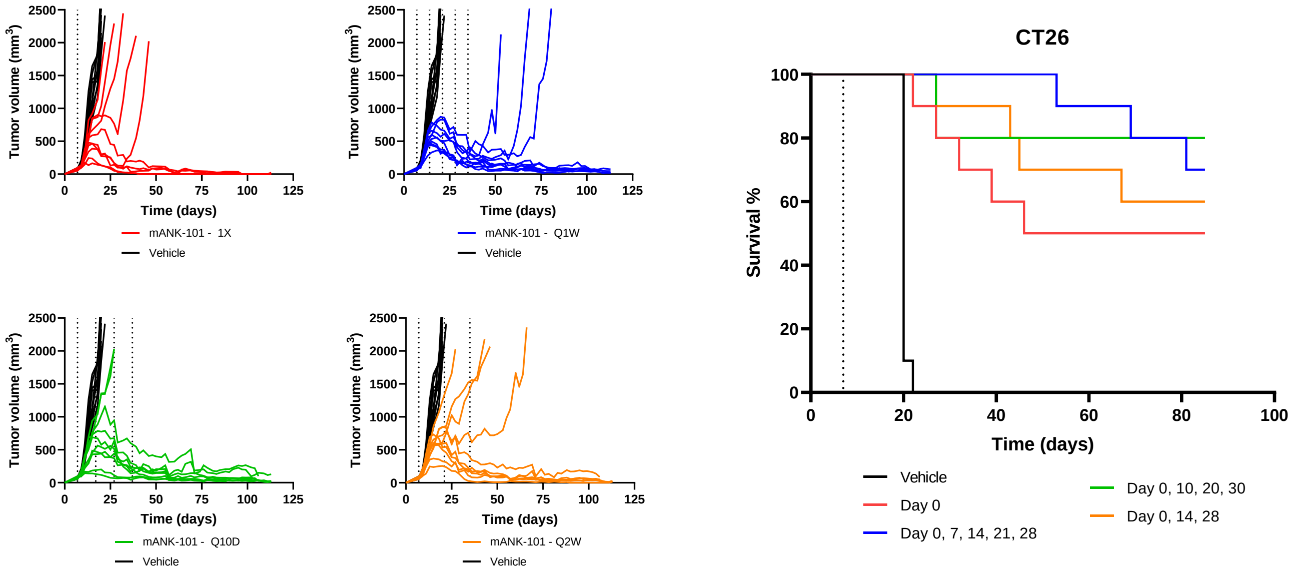

Supplemental Figure 3

D

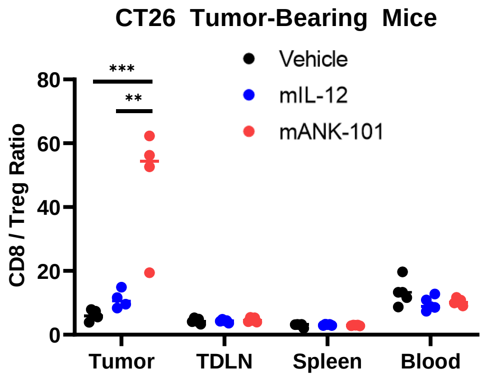

Supplemental Figure 4

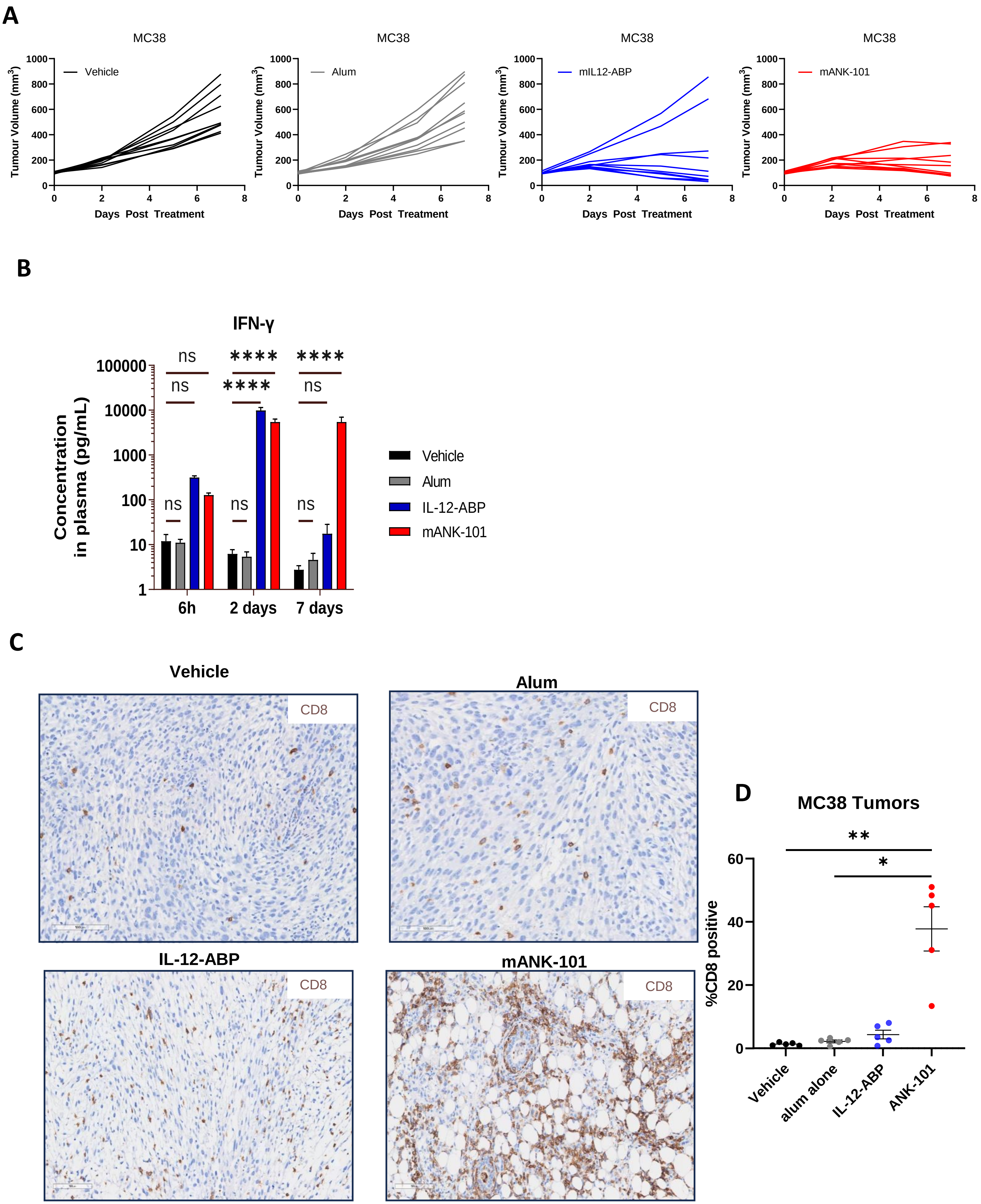

Supplemental Figure 5

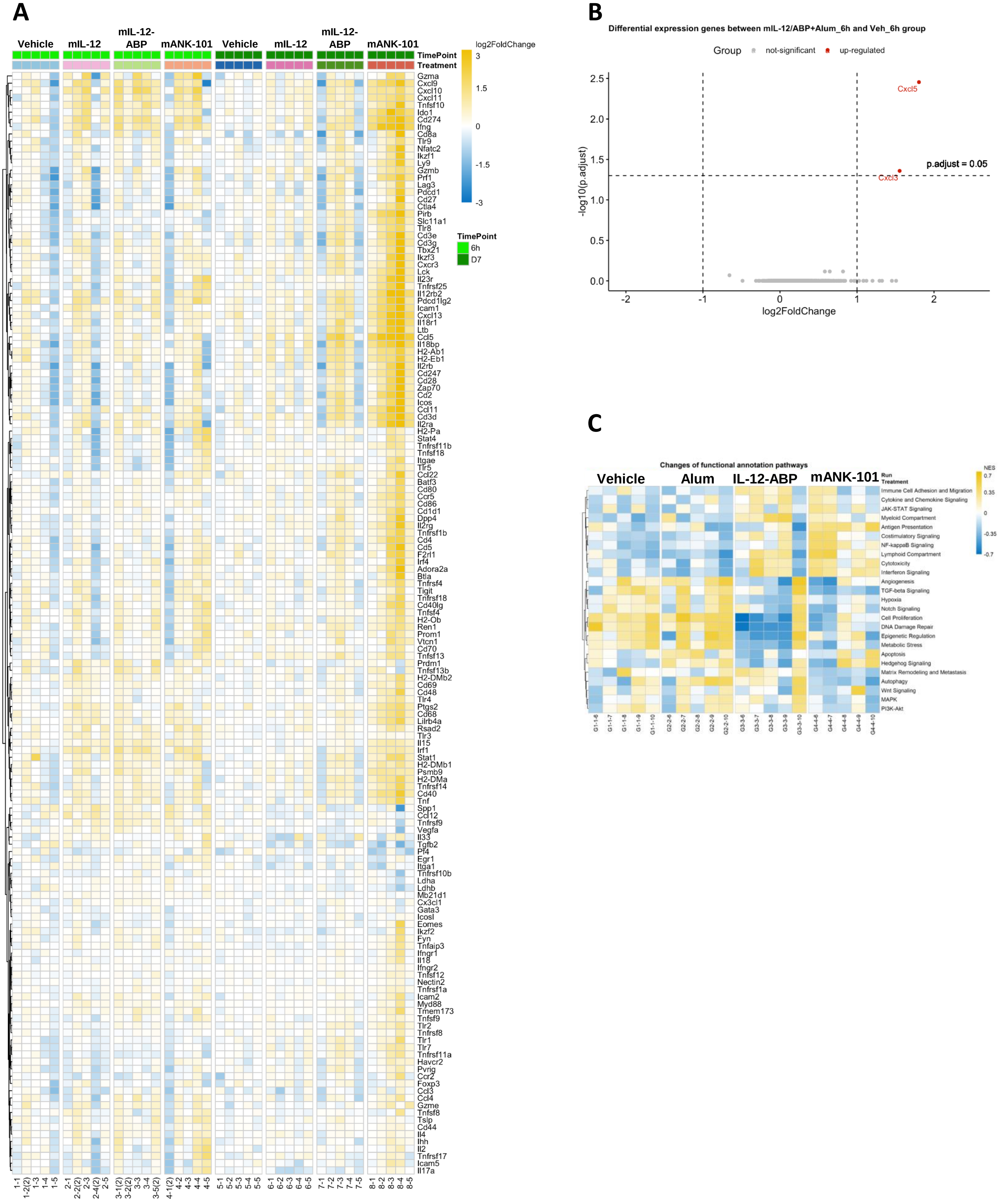

Supplemental Figure 6

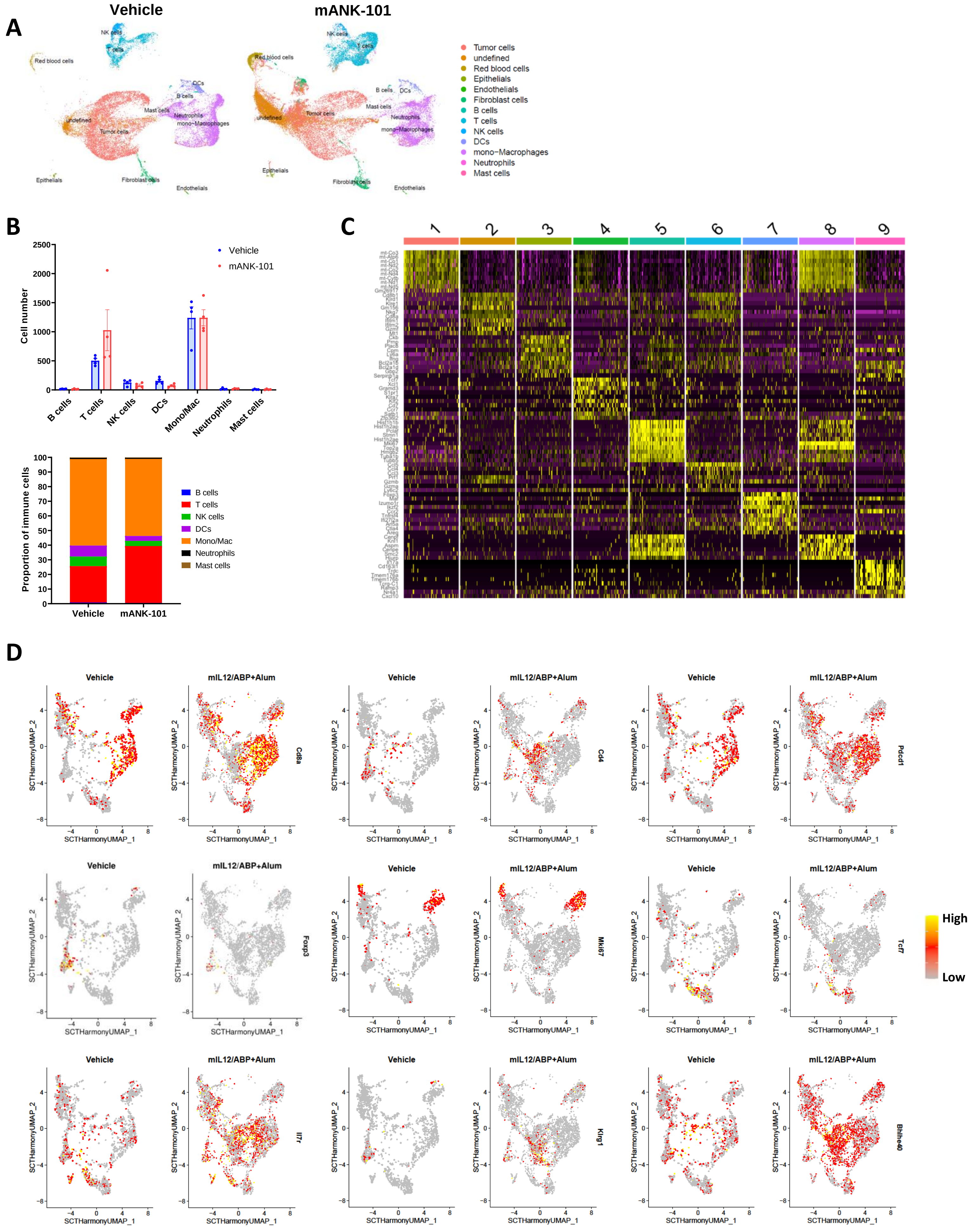

Supplemental Figure 7

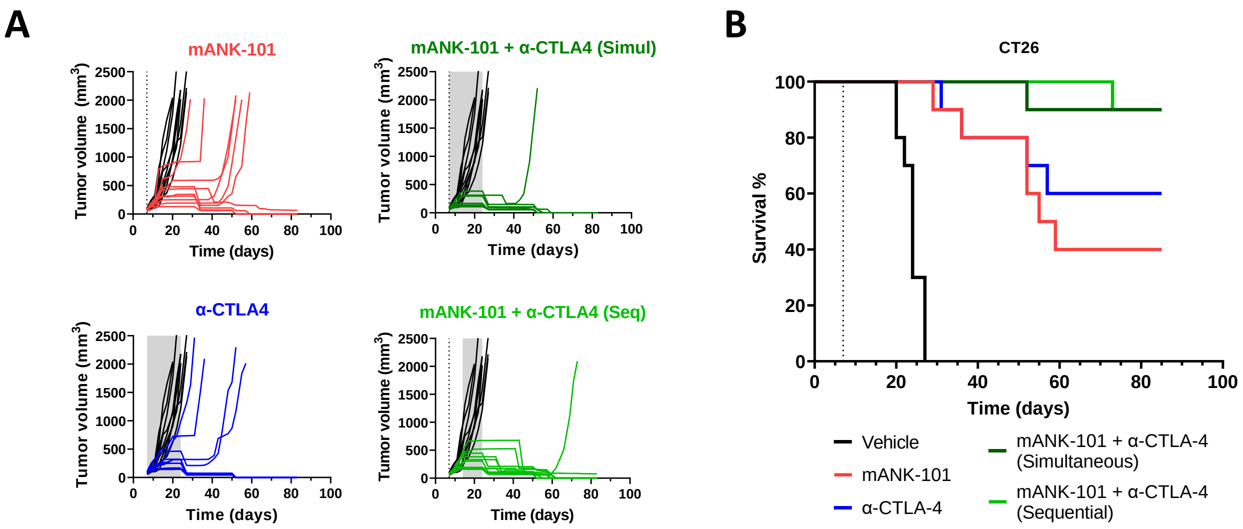

Supplemental Figure 8

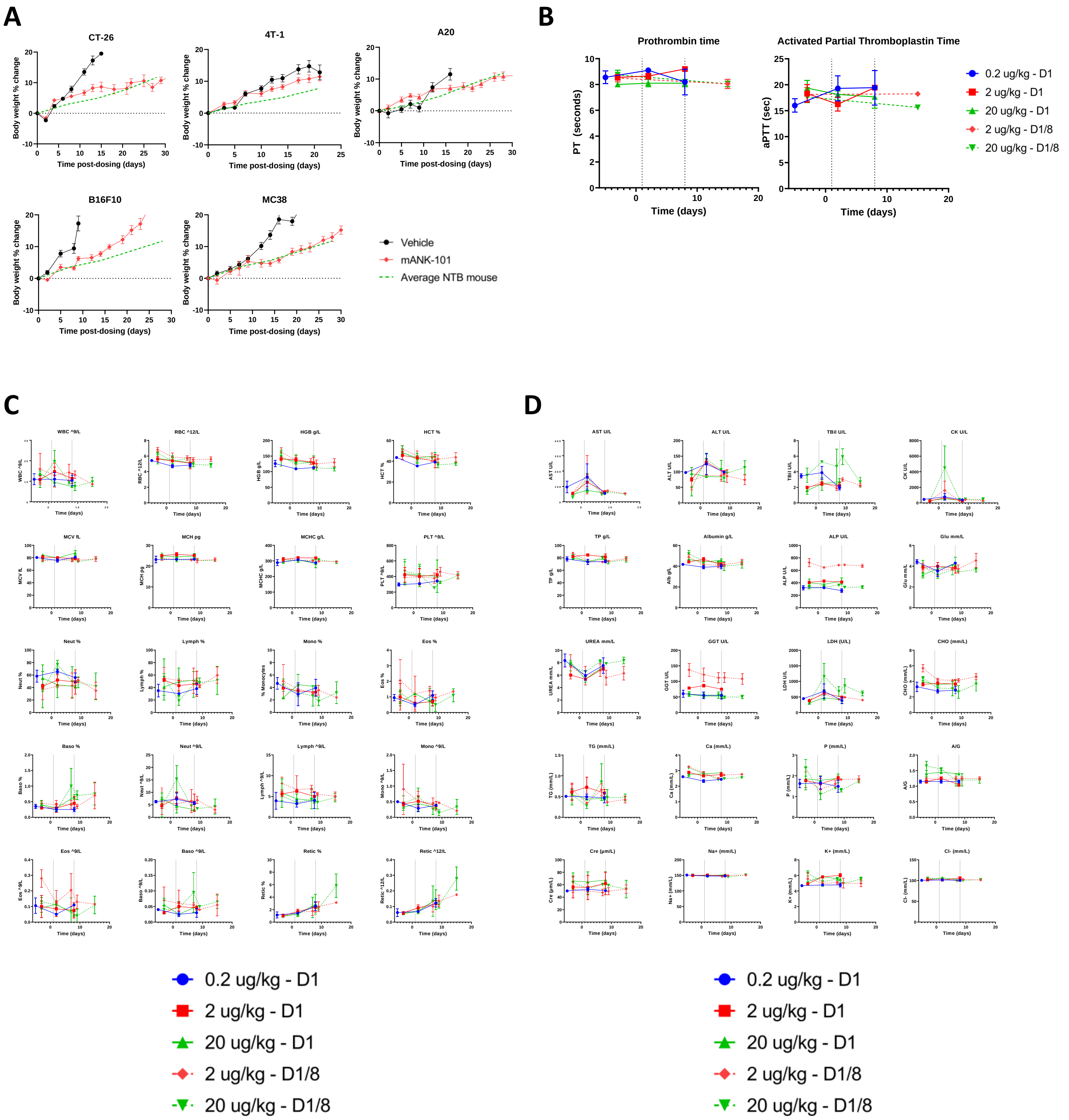

Table S1.

Construct	RT (mL)	Conductivity (mS/cm)
mIL-12	12.13	39.72
mIL-12-ABP	10.86	32.97
hIL-12	11.31	36.49
hIL-12-ABP	10.20	29.32

Table S2. Serum cytokines from cynomolgus macaques treated with ANK-101

Group	Animal ID	Dosing Day	Time	IFN-γ	IL-10	IL-12p70	IL-1β	IL-2	IL-6	IP-10	TNF-α
0.2 μg/kg Single dose	22-4451	D1	0h	BQL	BQL	BQL	BQL	0.646	2.20	130	BQL
			2h	BQL	BQL	BQL	BQL	0.758	1.94	151	BQL
			24h	6.61	BQL	1.88	BQL	1.01	1.48	298	BQL
			72h	8.61	BQL	2.79	BQL	1.02	1.21	324	BQL
			168h	6.41	BQL	BQL	BQL	0.956	2.45	138	BQL
	22-4452	D1	0h	BQL	BQL	BQL	BQL	0.602	3.52	64.8	BQL
			2h	BQL	BQL	BQL	BQL	BQL	3.51	59.1	BQL
			24h	BQL	BQL	BQL	BQL	BQL	3.34	107	BQL
			72h	BQL	BQL	BQL	2.15	0.838	12.9	277	BQL
			168h	BQL	BQL	BQL	BQL	0.897	5.80	252	BQL
2 μg/kg Single dose	22-4453	D1	0h	BQL	BQL	BQL	BQL	BQL	1.35	205	BQL
			2h	BQL	BQL	BQL	BQL	0.530	3.94	255	BQL
			24h	BQL	BQL	4.64	BQL	0.702	5.24	366	BQL
			72h	BQL	BQL	7.48	BQL	0.570	2.97	267	BQL
			168h	BQL	BQL	8.58	BQL	0.992	4.56	439	BQL
	22-4454	D1	0h	BQL	BQL	BQL	BQL	BQL	1.65	246	BQL
			2h	BQL	BQL	BQL	BQL	BQL	1.85	285	BQL
			24h	BQL	BQL	BQL	BQL	BQL	1.91	322	BQL
			72h	BQL	BQL	5.00	BQL	BQL	3.09	158	BQL
			168h	BQL	BQL	6.56	BQL	0.574	7.11	479	BQL
20 μg/kg Single dose	22-4455	D1	0h	BQL	BQL	BQL	BQL	0.566	0.894	356	BQL
			2h	BQL	BQL	BQL	BQL	0.626	1.06	354	BQL
			24h	BQL	BQL	4.62	BQL	0.642	4.44	571	BQL
			72h	BQL	BQL	11.4	BQL	0.586	1.18	267	BQL
			168h	BQL	BQL	45.5	BQL	0.964	3.70	1239	BQL
	22-4456	D1	0h	BQL	BQL	BQL	BQL	0.897	1.45	319	BQL
			2h	BQL	BQL	BQL	BQL	BQL	4.49	367	BQL
			24h	BQL	BQL	6.96	BQL	0.718	1.86	732	BQL
			72h	BQL	BQL	12.2	BQL	0.489	0.612	563	BQL
			168h	BQL	BQL	20.9	BQL	0.972	2.12	1211	BQL
2 μg/kg Repeat dose	22-4457	D1	0h	BQL	BQL	BQL	1.40	1.90	1.75	363	BQL
			2h	BQL	BQL	BQL	BQL	1.76	21.0	260	BQL
			24h	BQL	BQL	BQL	BQL	1.24	9.85	351	BQL
			72h	BQL	BQL	6.44	BQL	1.30	1.88	347	BQL
		D8	0h	BQL	BQL	12.0	BQL	1.10	1.41	509	BQL
			2h	BQL	BQL	11.2	BQL	1.09	2.94	514	BQL
			24h	BQL	BQL	11.1	BQL	0.913	2.23	513	BQL
			72h	BQL	BQL	23.5	BQL	1.27	2.06	498	BQL
			168h	6.79	BQL	4.24	BQL	1.44	3.69	374	BQL
	22-4459	D1	0h	BQL	BQL	BQL	BQL	1.71	1.29	423	BQL
			2h	BQL	BQL	BQL	BQL	1.41	9.26	322	BQL
			24h	BQL	BQL	4.65	BQL	0.992	21.9	690	BQL
			72h	BQL	BQL	7.68	BQL	1.26	1.65	312	BQL
		D8	0h	BQL	BQL	10.4	BQL	1.02	1.50	509	BQL
			2h	BQL	BQL	11.3	BQL	1.00	2.07	537	BQL
			24h	BQL	BQL	19.4	BQL	1.08	1.67	339	BQL
			72h	BQL	BQL	21.3	BQL	1.19	2.84	489	BQL
			168h	BQL	BQL	9.76	BQL	BQL	1.55	213	BQL
20 μg/kg Repeat dose	22-4458	D1	0h	BQL	BQL	BQL	BQL	0.809	0.886	225	BQL
			2h	BQL	BQL	BQL	1.75	0.798	12.9	142	BQL
			24h	BQL	BQL	12.9	BQL	0.916	7.85	1493	BQL
			72h	50.0	BQL	15.8	BQL	1.02	2.70	5608	BQL
		D8	0h	38.8	BQL	14.6	BQL	1.27	2.05	6519	BQL
			2h	47.3	BQL	15.2	BQL	1.21	6.77	7034	BQL
			24h	45.4	BQL	33.9	BQL	1.37	2.64	5180	BQL
			72h	398	BQL	40.1	BQL	1.33	2.42	8271	BQL
			168h	BQL	BQL	BQL	BQL	1.17	BQL	605	BQL
	22-4460	D1	0h	BQL	BQL	BQL	BQL	1.24	1.44	180	BQL
			2h	BQL	BQL	BQL	BQL	1.48	47.6	198	BQL
			24h	BQL	BQL	11.8	BQL	1.16	7.61	382	BQL
			72h	8.52	BQL	59.8	BQL	1.75	3.75	1303	BQL
		D8	0h	778	BQL	32.6	BQL	2.52	7.91	12631	BQL
			2h	625	BQL	29.9	BQL	2.32	14.0	12763	BQL
			24h	434	BQL	47.3	4.01	2.95	13.8	11244	BQL
			72h	16.0	BQL	43.7	BQL	3.52	14.7	8903	BQL
			168h	BQL	BQL	BQL	BQL	4.22	3.41	467	BQL

Table S3. SPECT/CT acquisition and reconstruction parameters

**SPECT Acquisition Parameters**	
System	NanoScan SPECT/CTTM (Mediso)
Scan range	Whole body
Scan duration & time points	30 m, 5 h, 24 h, 72 d, 10 d, 15 d, 21 d (30 minutes)
Energy window	28.37 20%FW keV
Number of projections	360
Time per projection	34 s
Pinhole aperture (# & diameter)	Aperture #63 (1.0 mm)
**SPECT Reconstruction Parameters**	
Smoothing	Low
Resolution	Medium
Iterations	Medium
Corrections	Attenuation and Scatter
Voxel size	1.0 × 1.0 mm

**CT Acquisition Parameters**	
System	NanoScan SPECT/CTTM (Mediso)
Scan range	Whole body
Tube voltage	50 kVp
Current	640 μA
Exposure time	300 ms
Number of projections	180
**CT Reconstruction Parameters**	
Algorithm	Filtered Backprojection
Filter	Cosine
Voxel size	0.250 × 0.250 mm

Table S4. FACS reagents

Marker	Fluorochrome	Clone	Cat.	Isotype	Vender
CD45	BV785	30-F11	103149	Rat IgG2b, κ	Biolegend
CD3	BUV395	17A2	740268	Rat IgG2b, κ	BD
CD4	BV421	GK1.5	100438	Rat IgG2b, κ	Biolegend
CD8	PE-eFluor610	53-6.7	61-0081-82	Rat IgG2a, κ	eBiosciences
Foxp3	PE	FJK-16s	12-5773-82	Rat IgG2a, κ	eBiosciences
CD335	BV711	29A1.4	137621	Rat IgG2a, κ	Biolegend
CD11b	BUV661	M1/70	612977	Rat IgG2b, k	BD
F4/80	BV510	BM8	123135	Rat IgG2a, κ	Biolegend
I-A/I-E	AF700	M5/114.15.2	107622	Rat IgG2b, κ	Biolegend
Ly-6G	BUV737	1A8	741813	Rat IgG2a, κ	BD
Ly-6C	FITC	HK1.4	128006	Rat IgG2c, κ	Biolegend
PD-1	BV650	J43	744546	Armenian Hamster IgG2, κ	BD
PD-L1	Percp-cy5.5	10F.9G2	124334	Rat IgG2b, κ	Biolegend
CD19	BV605	6D5	115540	Rat IgG2a, κ	Biolegend
CD86	PE-CY7	GL1	105014	Rat IgG2a, κ	Biolegend
CD103	APC	2E7	121414	Armenian Hamster IgG, κ	Biolegend
L/D	efluo780	NA	65-0865-14	NA	eBiosciences

Table S5. Genes used in Nanostring cell signatures

Cell type	Probe set
T cells	Cd3d, Cd3e, Cd3g, Cd6, Sh2d1a, Trat1
Macrophages	Cd163, Cd68, Cd84, Ms4a4a
Th1 cells	Tbx21
NK CD56dim cells	Il21r, Klr3dl1, Klr3dl2

Table S6. Hematology parameters measured in cynomolgus macaque study

Abbreviation	Parameter	Unit	Detection methods
WBC	Total leukocyte count	$\times 10^9$/L	The Basophil / Nuclear Lobularity Analysis Method+ Two-dimensional laser flow technology + Peroxidase staining method
Neut	Neutrophilic granulocyte	$\times 10^9$/L, %	Peroxidase staining method + The Basophil / Nucle ar Lobularity Analysis Method
Lymph	Lymphocyte	$\times 10^9$/L, %	Peroxidase staining method + The Basophil / Nuclear Lobularity Analysis Method
Mono	Mononuclear cell	$\times 10^9$/L, %	Peroxidase staining method + The Basophil / Nuclear Lobularity Analysis Method
Eos	Eosinophils	$\times 10^9$/L, %	Peroxidase staining method + The Basophil / Nuclear Lobularity Analysis Method
Baso	Basophilic cell	$\times 10^9$/L, %	Peroxidase staining method + The Basophil / Nuclear Lobularity Analysis Method
RBC	Erythrocyte count	$\times 10^{12}$/L	Two-dimensional laser flow technology
HGB	Hemoglobin	g/L	Total Hgb measured by cyanmethemoglobin
HCT	Hematocrit	%	Calculation based on RBC and MCV
Retic	Reticulocyte count	$\times 10^{12}$/L, %	Two-dimensional laser flow technology+ Fluorescent staining of nucleic acids
MCV	Mean corpuscular volume	fL	calculation
MCHC	Mean corpuscular hemoglobin conc.	g/L	calculation
MCH	Mean corpuscular hemoglobin	pg	calculation
PLT	Platelet	$\times 10^9$/L	Two-dimensional laser flow technology

Table S7. Clinical chemistry parameters measured in cynomolgus macaque study

Abbreviation	Parameter	Unit	Detection methods
ALT	Alanine aminotransferase	U/L	Alanine substrate method
AST	Aspartate aminotransferase	U/L	Aspartic acid substrate method
TP	Total protein	g/L	Biuret colorimrtry
Alb	Albumin	g/L	Bromocresol green method
A/G	Albumin/ globulin ratio	-	Calculated
TBil	Total bilirubin	μmol/L	Chemical oxidation
LDH	Lactate dehydrogenase	U/L	Lactic acid substrate method
ALP	Alkaline phosphatase	U/L	Kinetic method with NPP-AMP
CK	Creatine kinase	U/L	Creatine phosphocreatine substrate method
GGT	γ-glutamyl transpeptidase	U/L	GCANA substrate method
UREA	Urea	mmol/L	Urease glutamic dehydrogenase method
Cre	Creatinine	μmol/L	Sarcosine oxidase method
CHO	Total cholesterol	mmol/L	CHOD-PAP method
TG	Triglycerides	mmol/L	GPO-PAP method
Glu	Glucose	mmol/L	Hexokinase method
Ca	Calcium	mmol/L	Arsenazo III method
P	Phosphorus	mmol/L	Ultraviolet method
Na^+	Sodium ion	mmol/L	Electrode method
K^+	Potassium ion	mmol/L	Electrode method
Cl^-	Chloride ion	mmol/L	Electrode method
